# Putting Temperature and Oxygen Thresholds of Marine Animals in Context of Environmental Change: A Regional Perspective for the Scotian Shelf and Gulf of St. Lawrence

**DOI:** 10.1371/journal.pone.0167411

**Published:** 2016-12-20

**Authors:** Catherine E. Brennan, Hannah Blanchard, Katja Fennel

**Affiliations:** Department of Oceanography, Dalhousie University, Halifax, Nova Scotia, Canada; University of Connecticut, UNITED STATES

## Abstract

We conducted a literature review of reported temperature, salinity, pH, depth and oxygen preferences and thresholds of important marine species found in the Gulf of St. Lawrence and Scotian Shelf region. We classified 54 identified fishes and macroinvertebrates as important either because they support a commercial fishery, have threatened or at risk status, or meet one of the following criteria: bycatch, baitfish, invasive, vagrant, important for ecosystem energy transfer, or predators or prey of the above species. The compiled data allow an assessment of species-level impacts including physiological stress and mortality given predictions of future ocean physical and biogeochemical conditions. If an observed, multi-decadal oxygen trend on the central Scotian Shelf continues, a number of species will lose favorable oxygen conditions, experience oxygen-stress, or disappear due to insufficient oxygen in the coming half-century. Projected regional trends and natural variability are both large, and natural variability will act to alternately amplify and dampen anthropogenic changes. When estimates of variability are included with the trend, species encounter unfavourable oxygen conditions decades sooner. Finally, temperature and oxygen thresholds of adult Atlantic wolffish (*Anarhichas lupus*) and adult Atlantic cod (*Gadus morhua*) are assessed in the context of a potential future scenario derived from high-resolution ocean models for the central Scotian Shelf.

## Introduction

The global ocean has already incurred detectable changes in its heat and oxygen content, vertical stratification, pH, and sea level due to anthropogenic emissions of CO_2_ and other greenhouse gasses [1]. As greenhouse gas emissions are unlikely to decrease in the coming decades, changes in marine environmental conditions are expected to accelerate. With respect to coastal regions (defined here to be inclusive of continental shelves with water depths shallower than 200 m), significant challenges remain in observing, understanding, and projecting regional trends and processes. Predicting how marine species will respond to the changing environmental conditions is a major challenge that requires a regional perspective, since the physical and biochemical environment that a marine species encounters necessarily depends on the combination of influences affecting a given region. Physical and biochemical changes also need to be considered in the context of regional variability. Adding further complexity, marine species have a unique set of environmental preferences and requirements. As the ocean has warmed over recent decades, observations suggest that commercial fish species have not changed their preferred temperature ranges, but have instead altered their spatial distribution [2]. Fish are reportedly found at greater depths, and at more northerly locations [3]. Pinsky et al. [4] find shifts in species distribution occurring at different rates and directions within different coastal regions.

The northwest North Atlantic sustains a large fishing economy, and the Scotian Shelf has historically supported commercial groundfish, pelagic fish, and shellfish fisheries [5]. With the collapse of the Atlantic cod (*Gadus morhua*) fishery (Canada declared a moratorium in 1992) the eastern Scotian Shelf region has undergone a significant ecological shift from a large groundfish-dominated ecosystem to a system dominated by benthic macroinvertebrates, small groundfish and pelagic fish [6]. Since the 1970s, the average weight of individual fishes has decreased by 51% on the eastern shelf and 41% on the western shelf, with the greatest size declines associated with commercial fish species [7]. Fisheries observations indicate that fishing pressure also reduced the abundance of large-bodied predatory fish in the Gulf of St. Lawrence [7]. Despite these changes, the region remains one of the most important global fishing grounds: commercial fish landings from Atlantic Canada and Québec were valued at $2.4 billion in 2014 [8]. Understanding how marine species will respond to changing environmental conditions is therefore of great interest to various stakeholders ranging from marine managers and policy makers to coastal communities.

Each marine species has its preferred range of salinity, temperature, and oxygen values, and in most cases a total range outside of which the species does not thrive or survive. Numerous experimental studies have been performed over the last century to determine species- and life stage-specific environmental tolerances. While temperature and salinity tolerances have been widely studied, experiments involving oxygen and pH are relatively rare. Ekau et al. [9] reviewed oxygen thresholds and the associated impacts on the physiology, reproduction, growth, and mobility of pelagic species (N = 65) in the global ocean. Rogers et al. [10] compiled hypoxia tolerances of global freshwater and marine fish species (N = 151, of which 60% are marine species). Haigh et al. [11] evaluated the direct and indirect effects of ocean acidification on commercial marine species in the Northeast Pacific, and pointed to large knowledge gaps. Chabot et al. [12] identified experimental pH results for only three commercial species (cod, American lobster (*Homarus americanus*), and northern shrimp (*Pandalus borealis*)) in the northwest Atlantic region.

Based on a multi-model analysis under a business-as-usual emissions scenario, by year 2100 the northwestern North Atlantic is predicted to be strongly affected by multiple ecosystem stressors, including warming, acidification, oxygen depletion and decreased net primary production [13]. The identification of long-term temperature and oxygen trends (summarized below) indicates the occurrence of regional changes for the Scotian Shelf and Gulf of St. Lawrence. Long-term hydrographic trends in the northwest North Atlantic are overlain by marked decadal and longer-term variability associated with atmospheric oscillations; the dominant modes of this variability are the North Atlantic Oscillation (NAO) and the Atlantic Multidecadal Oscillation (AMO). The northwest North Atlantic is characterized by the most highly variable sea surface temperatures in the North Atlantic Ocean [14, 15].

Predicting how individual species may perform under changing environmental conditions is a complex problem. The variables are not independent: increases in temperature increase metabolic rates (and thus oxygen demand) and decrease oxygen solubility; when oxygen supply does not meet oxygen demand, the oxygen concentration constrains metabolic activity [16–19]. In a novel approach, Deutsch et al. [20] considered the combined effects of changing temperature and oxygen by constructing a metabolic index for 16 species utilizing the ratio of oxygen supply to resting metabolic oxygen demand. Changes in oxygen and pH both affect respiration in some marine species [21–24].

Additional uncertainty stems from species potential to adapt to changing environmental conditions (e.g. via acclimatization or genetic adaptation) [25, 26], and the indirect effects of environmental change on ecological communities, including changing species interactions as the geographic distributions and the timing of life events of species may shift relative to their predators, prey, competitors, etc. [27]. While species may be able adapt to changing environmental conditions via acclimatization or genetic adaptation, the adaptation potential can be diminished by high warming rates or the species current proximity to its thermal limit [25].

Here, we take a regional perspective in order to investigate the vulnerability of marine species to marine environmental change in the coastal northwest North Atlantic Ocean. Our approach includes a comprehensive literature survey to identify reported environmental preferences and requirements of marine fish and macroinvertebrate species that are central to the ecosystem in the Gulf of St. Lawrence and Scotian Shelf region of Atlantic Canada. The resulting dataset summarizes species- and life stage-specific temperature, salinity, pH, depth and oxygen preferences and thresholds of the identified 54 marine species. We then assess the vulnerability of a subset of those species to warming and oxygen depletion by comparing the determined temperature and oxygen ranges and thresholds to an observed trend, and include estimates of regional variability. Finally, we evaluate the environmental requirements of two vulnerable demersal species, adult Atlantic wolffish (*Anarhichas lupus*) and adult Atlantic cod, under a scenario of future temperature and oxygen changes derived from high-resolution ocean models for the central Scotian Shelf. Note that in this study we consider the environmental variables independently, and do not consider interactions (e.g. synergistic or antagonistic) between variables.

## Methods

### Study region hydrography, environmental variability and trends: Gulf of St. Lawrence and Scotian Shelf

**Fig 1:**
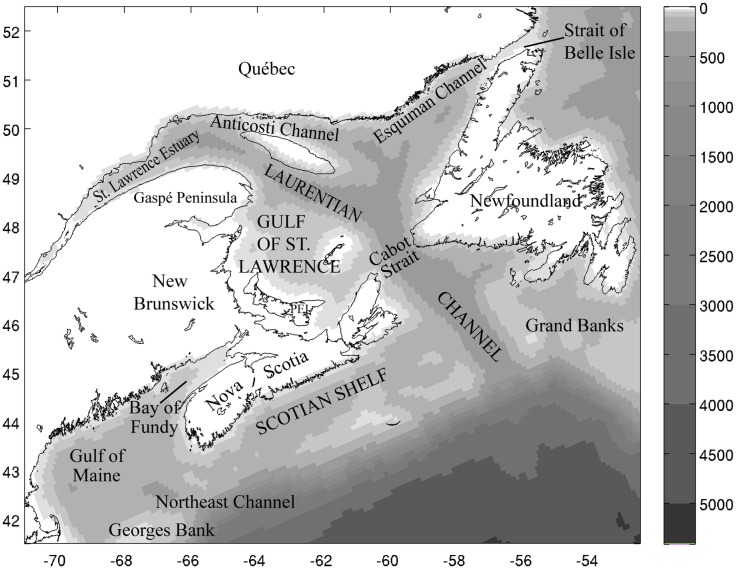
Map of the coastal northwest North Atlantic. Map of the coastal northwest North Atlantic between Georges Bank and Newfoundland. Bathymetry (depth in m), indicated by shading, is from the regional Atlantic Canada Model in ROMS of Brennan et al. [28].

We focus on the coastal northwest North Atlantic Ocean, extending from the Scotian Shelf into the Gulf of St. Lawrence (Fig 1; bathymetry is mapped from the Atlantic Canada Model in ROMS of Brennan et al. [28]). The Gulf of St. Lawrence is a 240,000 km^2^ semi-enclosed sea opening to the Atlantic Ocean at Cabot Strait and the Strait of Belle Isle. The Gulf is characterized by three deep channels: the Laurentian Channel extending 1,250 km to the St. Lawrence Estuary, the Esquiman Channel extending from the central Gulf towards the Strait of Belle Isle, and the Anticosti Channel north of Anticosti Island. The Scotian Shelf is the 96,0000 km^2^ section of the North American continental shelf off Nova Scotia and varies in width between 125 and 230 km. The Scotian Shelf is separated from the Grand Banks to the north by the Laurentian Channel, and from Georges Bank to the south by the Northeast Channel.

In the Gulf of St. Lawrence throughout most of the year, the Laurentian Channel consists of a shallow surface layer with seaward flow that shows large seasonal variations in temperature and salinity, a cold, medium-salinity intermediate layer, and an underlying warm and saline deep layer with landward flow [29]. Scotian Shelf waters are sourced from Gulf of St. Lawrence outflow, which turns southwestward onto the Scotian Shelf after leaving the Gulf (the Nova Scotia Coastal Current), the Labrador Current, which follows the shelf edge around the Grand Banks and along the Scotian Shelf, and the Gulf Stream, which sheds warm-core eddies that periodically impinge on the shelf. These three source waters have different temperature and salinity characteristics, which results in a distinct three-layer system in summer: a low-salinity surface layer with seasonal temperature variation, a cold, medium-salinity intermediate layer, and a warm, saline bottom layer [30]. In winter the upper two layers are homogenized due to surface cooling, convection and increased wind mixing resulting in a two-layer system.

Here we describe the regional variability (NAO and AMO) and the long term temperature and oxygen trends observed for the Scotian Shelf and Gulf of St. Lawrence. Trends are summarized in Table 1. The NAO is defined as the difference in atmospheric surface pressure between the Iceland low and the Azores high. Petrie [31] found NAO variability in the coastal northwest North Atlantic to be associated with differences in bottom temperature and salinity of up to 2°C and 0.4, respectively. During the negative (positive) NAO phase, warmer (colder) winters in the Labrador Sea result in a coherent warming (cooling) and increased (decreased) salinity in the Gulf of St. Lawrence and on the eastern Scotian Shelf. At the same time, an increased (decreased) Labrador Current transports cool and fresh (warm and saline) anomalies into the deep western Scotian Shelf. Over the last five decades, large negative phases dominated in the 1960s to early 1970s, followed by quasi-neutral phase values for a decade starting in the late 1970s, then large positive phases dominated from the late 1980s to 1995, and finally, small positive and negative values have occurred until present [32]. The AMO index is based on the spatially averaged, detrended and smoothed (e.g. 10-year running mean) North Atlantic Ocean sea surface temperature [33]. In contrast to the NAO, the AMO operates on much longer (65–80 year) timescales and is understood as a mode of Atlantic thermohaline circulation variability. The AMO is highly correlated with sea surface temperature (SST) anomalies in the Labrador Sea and east of Newfoundland, with the largest SST anomaly (∼0.3°C) found in the latter region [34]. A positive AMO phase began in the late 1990s (which can be thought of as supplemental warming on top of anthropogenic warming), and based on typical timescales could be expected to persist another two decades or so before a negative AMO cooling signature is again superimposed on anthropogenic warming.

**Table 1:** Observed regional trends in temperature and dissolved oxygen, and future climate scenarios. The latitude and longitude of long term monitoring sites include the central Scotian Shelf (43.84°N, 62.86°W), Cabot Strait (47.33°N, 59.77°W), and Lower St. Lawrence Estuary (48.73°N, 68.59°W).

Region	Sea Surface Temperature	Deep Water Temperature	Dissoved Oxygen
**Gulf of St. Lawrence**	+1.4°C (1985-2011) [36]	**Lower St. Lawrence Estuary: 48.73°N, 68.59°W, 320 m**	
		+1.65°C (1932-2003) [38]	−0.98 mmol m^−3^ yr^−1^ (1932-2003) [42]
		**Cabot Strait: 47.33°N, 59.77°W, 250 m**	
		+1.95°C (1932-2003) [38]	−0.88 mmol m^−3^ yr^−1^ (1960-2002) [42]
**Scotian Shelf**	+0.89°C (1982-2006) [35]	**Central Scotian Shelf: 43.84°N, 62.86°W, 150 m**	
			−1.06 mmol m^−3^ yr^−1^ (1961-1998) [42, 47]
		**Future Climate Scenarios, Central Scotian Shelf: 43.84°N, 62.86°W, 150 m**	
		Scen. I: Δ*T* = +6°C, Δ*S* = +0.5	Scen. I: Δ*O*_2_ = −12%
		Scen. II:—(Δ*T*, Δ*S* = 0)	Scen. II: Δ*O*_2_ = −21%
		Scen. III: Δ*T* = +6°C, Δ*S* = +0.5	Scen. IV: Δ*O*_2_ = −33%

Belkin [35] calculated a long term SST trend using annual and area averages over large marine ecosystems (LMEs) based on the Hadley Centre climatology (1°x1° spatial and monthly resolution) for 1957-2006, and for the warming period only (1982-2006). Between 1982 and 2006, SSTs increased on the Scotian Shelf by 0.89°C, and on the Newfoundland-Labrador Shelf (inclusive of the Gulf of St. Lawrence) by 1.04°C. These trends capture the combination of the NAO switch from large positive values in the late 1980s to negative and small positive values after 1995, together with the switch to positive AMO in the late 1990s. Based on an area-averaged time series of May-November SST from satellite data, Galbraith et al. [36] found Gulf of St. Lawrence SST increased by 1.4°C between 1985 and 2011 (an increase of 0.5°C per decade). They also found evidence of a mid-1990s temperature shift: the 1996-2010 average value is 1.1°C warmer than that from 1982-1993. Galbraith et al. [36] developed an air temperature proxy for Gulf of St. Lawrence SST, finding this region’s SST closely tracks air temperature. They calculated a 1.3°C air temperature increase from 1873-2011 (or 0.9°C per century ±0.3°C at 95% confidence intervals). Galbraith et al. [36] conclude that in the next 50 years, their calculated trend will result in Gulf of St. Lawrence SSTs higher than all previous records. The trend of 0.9°C per century is lower than the surface air temperature warming predicted over the next century by the IPCC of 2-3°C and 4-5°C, for RCPs 4.5 and 8.5, respectively [37], which takes into account nonlinear responses to current and future carbon emissions (using a multi-model ensemble average for the period 2081 to 2100, relative to the base period 1986-2005). The deep waters of the Gulf of St. Lawrence have also undergone warming: temperatures increased by 1.95°C at 250 m depth in the Cabot Strait and by 1.65°C at 320 m depth in the Lower St. Lawrence Estuary over the 1932-2003 period [38].

Exploring the magnitude of natural variability relative to long-term trends is of interest. In the Emerald Basin on the central Scotian Shelf, monthly temperature anomalies over four decades (1945-1992) from four depth levels between the surface and 150 m span 3-4°C, and the low frequency temperature anomalies are largest at depth (at 150 m) [14]. In addition to this large natural variability, predicted increases in surface air temperature for the 21st century are also large (i.e. 3°C increase in air temperature over the next century [37]). Natural variability will alternately dampen and amplify anthropogenic warming, suppressing the anthropogenic warming signal during some periods or driving it to even warmer extremes during others. In the near term, a positive AMO is likely to persist for the next couple of decades (amplifying the surface temperature trend), while the anticipated subsequent switch to a negative AMO will lessen the rate of warming in the region. If the 50-year trend of increasingly positive NAO indices (dampening the anthropogenic warming trend) continues (described in [32]), deeper waters in the Scotian Shelf region may tend towards cooler conditions. We note that surface warming combined with subsurface cooling will increase vertical stratification on the Scotian Shelf, affecting ventilation of oxygen to bottom waters.

Changes in the local oxygen budget must consider biology (oxygen is produced in the euphotic zone during photosynthesis, and consumed during respiration of organic matter), along with ocean physics (air-sea gas exchange of oxygen is affected via changes in oxygen solubility, dependent upon temperature and salinity), and changes in ocean circulation (either due to temperature-induced vertical stratification changes, or to density-driven transport changes) [39–41]. On the Scotian Shelf, oxygen concentrations at 150 m depth exhibited a long-term downward trend (−1.06 *μ*mol/L/yr) between 1961 and 1999 [42]. In the Gulf of St. Lawrence, bottom oxygen concentrations have declined over the past century, with the most significant change occurring in the bottom waters of the Lower St. Lawrence Estuary [43]. Gilbert et al. [42] estimated the oxygen trend at 320 m depth to be −0.98 *μ*mol/L/yr over the period 1932-2003. Two thirds of this oxygen decline is attributed to a warming of the bottom waters between 1932 and 2003, while the remaining oxygen decline is attributed to increased runoff nitrogen loads producing increased organic flux towards the bottom [38]. Gilbert et al. [42] additionally identified a negative oxygen trend at 250 m depth in the Cabot Strait, (−0.88 *μ*mol/L/yr, over the period 1960-2002).

Long-term trends (∼50 years, 1960-2009) in dissolved oxygen have recently been calculated by Stendardo and Gruber [32] for North Atlantic water masses adjacent to the shelf, including those influencing the Scotian Shelf and Gulf of St. Lawrence: the Labrador Sea (LS) to the north, the Newfoundland Basin (NFL) to the east of the Grand Banks, and the North American Basin (NAB) off the Scotian Shelf. The uppermost portion of the sampled water (i.e. between 100 m and ∼300 m depth) in all three regions is characterized by a negative trend in dissolved oxygen (see their Fig 3). Integrated over the 100-700 m depth, statistically significant oxygen trends are identified for NFL (−7.3±3.4*μ*mol/kg) and NAB (+4.4±3.8*μ*mol/kg) [32]. These off-shelf water masses hold the potential to influence the study region through exchange with shelf waters: both eddies and advection along submarine canyons transport slope waters onto the shelf [44].

We note the following observed pH trends: global ocean pH has decreased by approximately 0.1 over the past century [29], while in this region, pH has decreased by approximately 0.2 on the Scotian Shelf, with observations from the late 1990s to mid-2000’s ranging from above pH 8.3 to as low as pH 7.7 [45, 46]. Lower St. Lawrence Estuary bottom water pH has declined by 0.2 to 0.3 [29], which is attributed to increased organic matter fluxes.

### Literature review: Inclusion criteria and species selection

For a species to be included in the literature review, we required that it meets at least one of the following eight criteria: species with commercial fisheries, species at risk, bycatch species, baitfish species, invasive species, vagrant species (species outside their normal range), species important for energy transfer in the ecosystem, and predator or prey of the above species. We additionally required that oxygen information be available for each species. We excluded marine mammals and reptiles (which are not directly affected by low seawater dissolved oxygen since they breath air at the surface).

Commercial species are defined to be those for which a Canadian nominal catch value is provided by the Department of Fisheries and Oceans Canada (DFO) in 2014 (the most recent data readily available). Species at risk are defined as those listed by the Committee on the Status of Endangered Wildlife in Canada (COSEWIC), or those listed on Canada’s Species at Risk Act (SARA) Public Registry [48]. Commercial species and species at risk account for most of the species in our review, but we include species that fit the remainder of our criteria if information was available.

The literature review was completed between May and August 2013, with article publication dates ranging from 1930 to 2013. Many of the publications used were those cited in Ekau et al. [9], and additional articles were found using the databases Web of Science, Google Scholar, and Novanet with search terms “marine hypoxia”, “[Latin or common name] + oxygen levels”, or “[Latin or common name] + hypoxia” (e.g., “Anarhichas lupus oxygen levels”).

Of the Scotian Shelf and Gulf of St. Lawrence marine fishes and macroinvertebrates meeting the inclusion criteria and for which oxygen level information is available, 54 species are identified in Table 2, each with its relevant criteria. Although species inhabiting the Scotian Shelf and Gulf of St. Lawrence region are the focus of our study, we additionally include *Mnemiopsis leidyi* (the sea walnut), a notoriously invasive species with a wide range of environmental tolerances [49], as it could potentially invade the study region from the south in the future. The various life stages of each species (e.g. larval winter flounder (*Pseudopleuronectes americanus*) or adult Atlantic herring (*Clupea harengus*), herein referred to as lifestage-species) for which we report environmental ranges are characterized as belonging to one of four vertical habitat zones: pelagic, demersal, benthic, or infaunal. Pelagic organisms occupy the middle or surface ocean waters uninfluenced by the shore or bottom; demersal species live on or near the seabed; benthic creatures live on the seabed; and infauna live within bottom substrate.

**Table 2:** List of selected species (N = 54). Latin name, common name, and inclusion criteria are provided. BC = bycatch, BF = baitfish, CF = commercial fishery, CFP = predator/prey of commercial fishery species, E = endangered, EET = ecosystem energy transfer, IS = invasive species, SC = special concern, T = threatened, VS = vagrant species. Commercial values based on total Canadian fishery Atlantic coast landings values in 2014 (DFO, 2016).

Latin name	Common name	Criteria
*Acipenser oxyrinchus*	Atlantic sturgeon	COSEWIC ‘T’^1^
*Alosa aestivalis*	Blueback herring	CF $39.4M^3^^a^
*Alosa pseudoharengus*	Alewife	CF $1.3M^3^
*Alosa sapidissima*	American shad	CF $21K^3^
*Anarhichas lupus*	Atlantic wolffish	COSEWIC/SARA ‘SC’^1^
*Anarhichas minor*	Spotted wolffish	COSEWIC/SARA ‘SC’^1^
*Apeltes quadracus*	Fourspine stickleback	BF^2^
*Arctica islandica*	Ocean quahog	CF $48.5M^3^^b^
*Brevoortia tyrannus*	Atlantic menhaden	BC^3^
*Callinectes sapidus*	Blue crab	Striped bass prey^4^
*Cancer irroratus*	Rock crab	CF $6.3M^3^^c^
*Carcinus maenas*	Green crab	IS^5^
*Chionoecetes opilio*	Snow crab	CF $533.7M^3^
*Clupea harengus*	Atlantic herring	CF $39.4M^3^^a^
*Crangon septemspinosa*	Sand shrimp	CFP (winter flounder predator)^6^
*Crassostrea virginica*	American oyster	CF $4.0M^3^
*Cyclopterus lumpus*	Lumpfish	CF $110K^3^
*Cynoscion regalis*	Weakfish	VS^2^
*Dyspanopeus sayi*	Say’s mud crab	CFP (oyster/quahog predator)^7^
*Fundulus heteroclitus*	Mummichog	BF^2^
*Gadus morhua*	Atlantic cod	COSEWIC ‘E’, CF $17.5M^1^,^3^
*Gadus ogac*	Greenland cod	CFP (Greenland halibut predator)^2^
*Glyptocephalus cynoglossus*	Witch flounder	CF $14.6M^3^^d^
*Hippoglossus hippoglossus*	Atlantic halibut	CF $43.2M^3^
*Homarus americanus*	American lobster	CF $942.2M^3^
*Hyperoglyphe perciformis*	Rudder-fish	VS^2^
*Ilyanassa obsoleta*	Eastern mudsnail	EET^8^
*Mercenaria mercenaria*	Northern quahog (or Hard clam)	CF $48.5M^3^^b^
*Merluccius bilinearis*	Silver hake	CF $7.0M^3^^e^
*Mnemiopsis leidyi*	Sea walnut	IS^9^
*Morone saxatilis*	Striped bass	COSEWIC E/SC^1^
*Mugil cephalus*	Mullet	VS^2^
*Mya arenaria*	Soft-shell clam	CF $48.5M^3^^b^
*Mytilus edulis*	Blue mussel	CF $3K^3^
*Palaemonetes pugio*	Daggerblade grass shrimp	EET^10^
*Palaemonetes vulgaris*	Marsh grass shrimp	EET^10^
*Pandalus borealis*	Northern shrimp	$369.1M^3^
*Paralichthys dentatus*	Summer flounder	VS^2^
*Peprilus triacanthus*	Butterfish	BC^3^
*Placopecten magellanicus*	Deep sea scallop	CF $177.8M^3^
*Prionotus carolinus*	Northern sea robin	VS^2^
*Pseudopleuronectes americanus*	Winter flounder	CF $14.6M^3^^d^
*Raja erinacea*	Little skate	BC^2^
*Reinhardtius hippoglossoides*	Greenland halibut	CF $64M^3^
*Rhithropanopeus harrisii*	North American mud crab	IS^13^
*Salmo salar*	Atlantic salmon	COSEWIC ‘SC’^1^
*Scomber scombrus*	Atlantic mackerel	CF $5.0M^3^
*Scophthalmus aquosus*	Windowpane	BC^14^
*Sphoeroides maculatus*	Northern puffer	VS^2^
*Spisula solidissima*	Atlantic surfclam	CF $48.5M^3^^b^
*Streblospio benedicti*	Spionid polychaete	Grass shrimp prey^11^
*Tautoga onitis*	Tautog	CFP^12^
*Tautogolabrus adapersus*	Cunner	BC^2^
*Urophycis chuss*	Red hake	CF $7.0M^3^^e^

^1^[48]

^2^[30]

^3^[8]

^4^[50]

^5^[51]

^6^[52]

^7^[53]

^8^[54]

^9^[55]

^10^[56]

^11^[57]

^12^[58]

^13^[59]

^14^[60, 61]

^a^Reported landings value corresponds to category “Herring”

^b^Reported landings value corresponds to category “Clams/Quahaug”

^c^Reported landings value corresponds to category “Crab, Other”.

^d^Reported landings value corresponds to category “Flatfishes”

^e^Reported landings value corresponds to category “Hake”.

### Literature review: Environmental categories

We surveyed the literature for species-level information on temperature, salinity, pH, depth, and dissolved oxygen. For oxygen, four categories were found in the literature: (i.) preferred level, (ii.) critical level, (iii.) median lethal level, and (iv.) level of no survival. As no formal definition exists for the preferred oxygen category, we interpret it as the range of dissolved oxygen concentrations for which an individual species experiences no adverse effects. For the preferred oxygen level, a single value indicates that only the minimum preferred oxygen concentration is known. This preferred oxygen category encompasses other terms occasionally used in the literature such as “*suitable*” and “*optimal*” oxygen (e.g. [50, 62, 63]). In the literature, the term critical oxygen level was either undefined, or used to describe an oxygen concentration at which an organism experienced either visible physical changes or measurable changes in its chemical physiology, with the most common definition describing an oxygen level that does not cover the energy demand of an organism’s aerobic metabolic pathways (e.g., [9, 64] in [10, 65–67]. We interpreted this suite of definitions to describe an oxygen level at which oxygen consumption becomes dependent on oxygen concentration, below which point the metabolism slows. The median lethal level is the statistically determined value at which 50% of the individuals in a study die [68]; the level of no survival corresponds to 100% mortality.

For each species, we summarize oxygen information (S1 Table) including the identified preferred oxygen, critical oxygen, median lethal and no survival oxygen values (or ranges) in units of mg/L (unit conversion is described in the table notes). Latin name and common name are specified, along with the water column habitat zone (pelagic, demersal, benthic, and infaunal), and if described, the life stage under study (adult, juvenile, larval, egg, etc.). For many of the species, environmental preferences and requirements are listed for multiple life stages. We provide the original reference and the original oxygen values and units, and categorize the evidence as experimental or observational. We include additional notes to indicate experimental conditions, duration, and the species response when oxygen concentrations fall below the specified oxygen level.

We list the temperature, salinity, and depth preferences and requirements for each species. Species or life stages with insufficient information are highlighted in red with the annotation ‘*no data available*’. Four temperature categories are reported from the literature (S2 Table): total temperature range, preferred temperature range, lower and upper intolerant temperatures, and lower and upper lethal temperatures. Total and preferred temperature ranges are most commonly reported in the literature. The salinity categories are the same as the temperature categories (S3 Table). Three depth categories are reported from the literature (S4 Table): total depth range, mean depth, and preferred depth.

We report the pH information identified for Scotian Shelf and Gulf of St. Lawrence marine species in S5 Table. pH information type (observational or experimental) is indicated in the description, along with which response variable (e.g. growth rate, calcification rate, etc.) was measured. We list the pH levels tested in each experiment, along with the corresponding observed result (0 = no impact, 1 = decrease, 2 = increase). pH effects were measured for 9 species across 10 studies, for multiple response variables, including hatching success, survival, otolith size, final weight, growth rate, swimming performance, length of intermoult interval, calcification rate, development rate (e.g. progression between life stages), size, settlement success, feeding efficiency, and shell extension rate.

### Regional hydrographic trends and variability in context of species requirements

The compiled environmental tolerances and thresholds can serve to characterize species vulnerability when evaluated with respect to current or potential future environmental conditions. We evaluate species vulnerability to environmental changes on the central Scotian Shelf by comparing a subset of species requirements against an observed multidecadal oxygen trend, estimates of oxygen variability, and a set of future scenarios for temperature and oxygen. We selected a benthic species (snow crab, *Chionoecetes opilio*), three demersal fishes (adult Atlantic wolffish, adult Atlantic cod, and witch flounder, *Glyptocephalus cynoglossus*), and one pelagic fish species (adult Atlantic salmon, *Salmo salar*) for comparison. The selected species are distributed over the Scotian Shelf with depth ranges that intersect 150 m, and may be vulnerable to environmental change. Three of these species have relatively high critical oxygen levels (e.g. adult Atlantic wolffish, adult Atlantic salmon, adult Atlantic cod have critical oxygen levels of 5.69-6.64 mg/l (K. Gamperl, pers. comm.), 4-5 mg/l [66, 69], and 2.9-3.19 mg/l [70], respectively), and snow crab has a restricted thermal range (−1 to 4°C [71]). Witch flounder has the highest no survival oxygen level (0.97-2.15 mg/l [72]) within the demersal species category. Atlantic wolffish is a species of special concern with both COSEWIC and SARA listings, while COSEWIC lists Atlantic cod and Atlantic salmon as endangered and special concern, respectively.

To characterize Scotian Shelf bottom oxygen and temperature, we utilize estimates of oxygen and temperature variability from recent observations from Ocean Tracking Network (OTN) benthic pods, and an historical time series of annual oxygen anomalies spanning four decades [47]. We determined high-frequency variability with ∼hourly observations from OTN benthic pods deployed on the Halifax Line at four stations: HFX008 (63.49°W, 44.44°N) at 60 m, HFX028 (63.36°W, 44.32°N) at 115 m, HFX069 (63.10°W, 44.09°N) at 160 m, and HFX097 (63.19°W, 43.91°N) at 133 m (see benthic pod locations on inset map of Fig 4). The benthic pods recorded conductivity, temperature, and dissolved oxygen over the period November 2011 to April 2015 using a Sea-Bird Electronics 37-SIP MicroCAT and an Aanderaa sensor (respectively; data available at http://gliders.oceantrack.org/data/pod/). We quality controlled the benthic pod data, by removing outliers and comparing values against independent measurements of temperature and oxygen from nearby glider tracks. We estimated interannual variability using a detrended historical 38-year (1961-1999) time series of annual oxygen anomalies from Emerald Basin on the central Scotian Shelf (43.84°N, 62.86°W) at 150 m depth from Petrie and Yeats (2000). The standard deviation of the detrended historical time series is 0.71 mg/l. We interpolated between benthic pod stations HFX097 (133 m) and HFX069 (160 m) to 150 m, and we determined the mean oxygen content at 150 m is 4.32 mg/l (standard deviation *σ* = 0.36 mg/l) based on benthic pod data.

In addition to utilizing observed trends of temperature and oxygen, model scenarios can be employed to represent potential changes for the Scotian Shelf and Gulf of St. Lawrence region. Recent high-resolution modelling studies have produced temperature and/or oxygen predictions over the Scotian Shelf [73, 74]. We analyze the scenarios of future change simulated for the central Scotian Shelf at 150 m, summarized in Table 1. Scenario I is based on a 2xCO2 experiment wherein atmospheric CO_2_ increases by 1 percent annually until reaching a doubled atmospheric concentration) utilizing a high resolution global model (GFDL CM2.6 with 0.1° horizontal resolution ocean model). CO_2_ doubling occurs in the simulation after 70 years, and large temperature and salinity increases occur in shelf bottom waters (approximately 6°C and 0.5 psu). Saba et al. [74] associate these changes with a northward shift of the Gulf Stream. We assign a 12 percent oxygen decrease to Scenario I, which is the reduction in oxygen solubility for the simulated warming and salinity increase. Scenario II uses a high-resolution regional biophysical ocean model (Atlantic Canada Model, or ACM, described in Brennan et al. [28]), and simulates the effect of lower oxygen in off-shelf slope water on Scotian Shelf bottom water. This experiment illustrates how source water deoxygenation alone can influence Scotian Shelf bottom water: a 21 percent oxygen decrease due to the deoxygenation of off-shelf deep Atlantic Basin water. Scenario III is the combination of Scenarios I and II, and includes both reduced oxygen in Atlantic off-shelf slope water [73], and the oxygen solubility decrease resulting from large temperature and salinity increases (6°C and 0.5, respectively) in shelf bottom waters [74]. Scotian Shelf bottom oxygen is reduced by 33 percent in Scenario III.

The environmental requirements compiled from the literature survey may be employed to evaluate species vulnerability in the context of observed trends and variability or future scenarios. We construct a potential future central Scotian Shelf state that regional species could encounter by adding the Scenario III temperature and oxygen changes to the present conditions observed at OTN benthic pods (represented by mean and ±2 standard deviations from data recorded at HFX008 at 60 m, HFX028 at 115 m, and HFX069 at 160 m—we neglect HFX097 at 133 m given it has very similar thermal and oxygen conditions to HFX069). Note that this construction thus assumes future variability can be represented by present day observed variability.

## Results

### Literature review: Compilation of environmental requirements

Oxygen information identified in the literature for each species is summarized in S1 Table (supplementary material), and temperature, salinity, and depth preferences and requirements are listed for each species (when available) (S2–S4 Tables). The pH information available for the listed species in the study region is presented in S5 Table.

**Fig 2:**
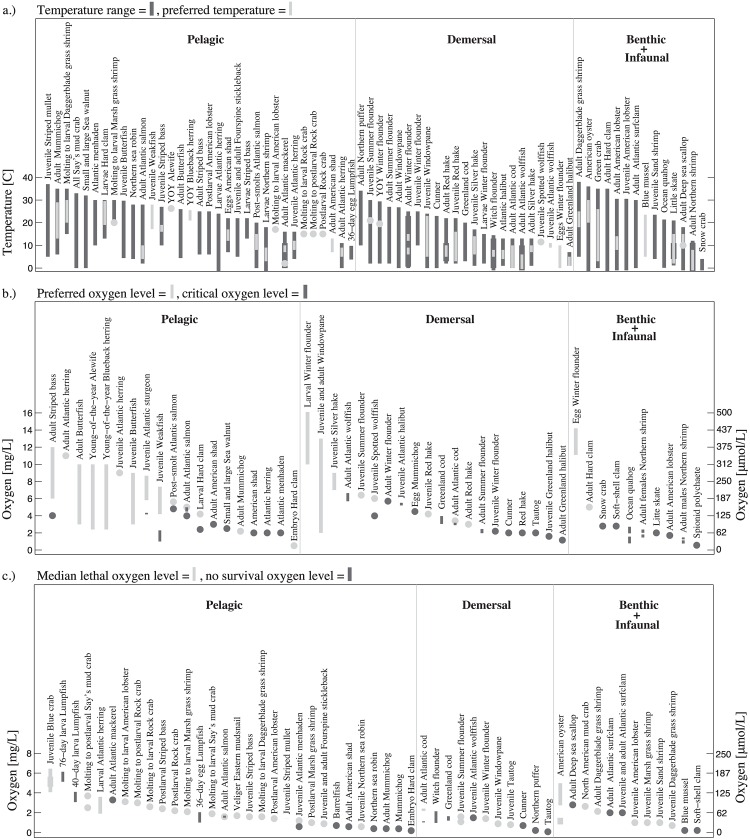
Temperature and oxygen ranges and thresholds for species identified in Table 2. Quantities include (a.) temperature range and preferred temperature, and levels of (b.) preferred oxygen (range shown as light gray bars and single values shown as filled circles), critical oxygen (dark gray), and (c.) median lethal oxygen (50% mortality) (light gray), and no survival oxygen (100% mortality) (dark gray). Species are divided (from left to right) into pelagic, demersal, and benthic/infaunal, in the order of maximum oxygen value.

Temperature ranges are defined by the upper and lower thermal limits of a given species’ habitat. These are plotted as the dark gray bars in Fig 2a (thick bars indicate multiple references). The temperature a species prefers to inhabit is given as either one value (light gray circle; typically the optimal value), or a preferred temperature range (light gray bar). The species are divided (from left to right) into pelagic, demersal, and bethic/infaunal, and are presented in order of maximum temperature value (for 68 lifestage-species along the x-axis).

The adult hard clam, *Mercenaria mercenaria*, American oyster, *Crassostrea virginica*, and green crab, *Carcinus maenas*, tolerate the widest temperature range (−6 to 35°C, −1.7 to 36°C, and 0 to 35°C, respectively), while snow crab has the narrowest temperature range (−1 to 4°C). The green crab is a notoriously invasive species [51], and its eurythermic temperature tolerance may allow it to expand further as the ocean warms. The green crab was not present in northern Nova Scotia until the 1980s, a time associated with increasing sea surface temperatures on the Scotian Shelf [75].

Adult hard clam tolerates the most extreme cold temperatures, and daggerblade grass shrimp, *Palaemonetes pugio*, the highest temperatures (up to 38°C). Species with the most narrow temperature ranges tend to be cold-water inhabitants (e.g. snow crab, adult Greenland halibut, *Reinhardtius hippoglossoides*, lumpfish 36-day egg, *Cyclopterus lumpus*). Eggs, larvae, and juveniles typically require warmer temperatures than later life stages, as exemplified by American shad, *Alosa sapidissima*, Atlantic wolffish, Atlantic herring, mummichog, *Fundulus heteroclitus*, American lobster, hard clam, silver hake, *Merluccius bilinearis*, striped bass, *Morone saxatilis*, daggerblade grass shrimp, winter flounder, and Atlantic salmon (S2 Table).

As for preferred temperatures, the green crab has the widest range (3–26°C), while the narrowest ranges span only 1 to 2°C and include witch flounder (5 to 7°C and 7 to 8°C based on two different studies), winter flounder egg (3 to 5°C), and juvenile Atlantic wolffish (9 to 11°C) (Fig 2a, S2 Table). American oysters and molting to larval daggerblade grass shrimp prefer the warmest temperatures (20 to 30°C). Adult northern shrimp, *Pandalus borealis*, and adult Greenland halibut prefer the coolest temperatures (−1 to 8°C and 0 to 5°C are the two preferred ranges found for the former, and 1 to 5°C for the latter).

Trends in both age and size are found in the temperature preferences. Juveniles of many fish species prefer warmer temperatures than adults [76], which is supported by this literature survey: eggs, larvae, and juveniles of most marine species prefer warmer temperatures compared to later life stages (Fig 2a). As for fish size, increased fish size is associated with lower temperature optima [77], as displayed in Atlantic cod, Greenland halibut, and spotted wolffish, *Anarhichas minor*, among others.

Individual lifestage-species’ preferred oxygen levels are plotted, along with the critical oxygen level, in Fig 2b. The median lethal and no survival oxygen levels are plotted in Fig 2c. The oxygen requirements for species in the region range from 0 mg/L (American oyster median lethal level) to 6.64 mg/L (adult Atlantic wolffish upper critical level) (Fig 2b and 2c, S1 Table). While the demersal Atlantic wolffish has the highest critical oxygen value (5.69 to 6.64 mg/L), the infaunal spionid polychaete, *Streblospio benedicti*, has the lowest (0.57 mg/L). The three highest critical values belong to demersal fish (ranging from 5.39 to 6.64 mg/L). Adult and post-smolt salmon have the highest pelagic critical oxygen levels (4-5 mg/L and 4.82 mg/L, respectively). The highest median lethal oxygen level is that of the juvenile blue crab, *Callinectes sapidus* (4.08 to 6.44 mg/L). Demersal fish do not appear to have lower oxygen ranges than pelagic fish (demersal fish have fairly low median lethal levels of 0.9 to 2.4 mg/L), but since the bottom of the water column generally contains less oxygen [9], extensive demersal fish declines can occur in areas with low bottom oxygen [78, 79].

**Fig 3:**
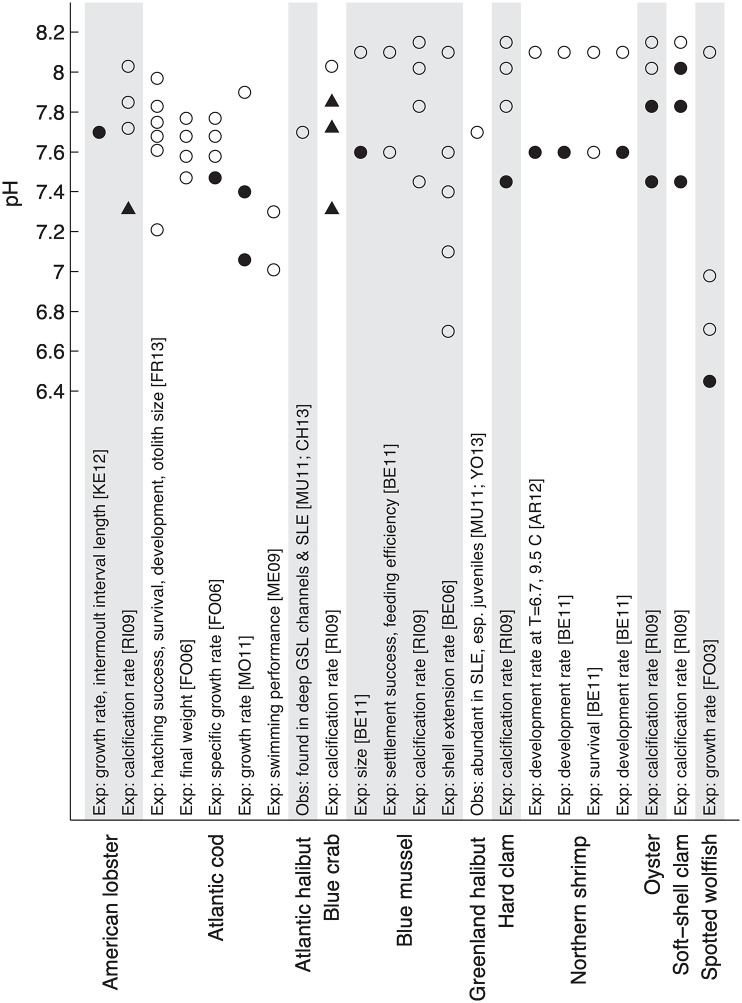
pH information for species identified in Table 2. Text indicates whether information originates from experimental (Exp.) or observational (Obs.) studies, the variable(s) observed (e.g. calcification), and the reference(s). No impact to the species at the indicated pH level is represented by open circles, while an observed impact is represented as a decrease (filled circle) or increase (filled triangle). Note that the AR12 Northern shrimp experiments were conducted at two temperatures (6.7 and 9.5 C), with a decreased development rate observed only at the lower temperature. References are coded as follows: AR12 = [80]; BE06 = [81]; BE11 = [82]; CH13 = [12]; FO03 = [83]; FO06 = [84] (FO06 did not maintain constant pH: here, time mean pH values are plotted); FR13 = [85]; KE12 = [86]; ME09 = [87]; MO11 = [88]; MU11 = [29]; RI09 = [89]; YO13 = [90]. GSL = Gulf of St. Lawrence; SLE = St. Lawrence Estuary.

Increased dissolved carbon dioxide can affect respiration in large invertebrates and fish (whereby internal acidification depresses cellular and respiratory activity as well as protein synthesis), and can lead to decreased calcification rates or shell dissolution in calcifying organisms (e.g. clams and mussels) [45]. Experimental results for the effects of lowered pH on species are both diverse (multiple impact types were evaluated) and scant. Several species included in S5 Table (and shown in Fig 3) exhibited responses above pH 7.6 in experiments, including American lobster, blue crab, oyster, and soft-shell clam, *Mya arenaria*.

Of the nine species investigated via experimental studies, low pH may cause the highest-impact responses in the soft-shell clam and oyster, which experienced reduced calcification rates at relatively high pH levels. Moderate-impact responses were identified for the hard clam (reduced calcification rate at ∼7.4), northern shrimp (at pH ∼7.6, development time slowed, which could expose zoeal stages to increased predation), and American lobster (decreased growth rate and a longer inter moult interval at pH ∼7.7, with increased calcification rate at ∼7.3). Low-impact responses were found for the Atlantic cod (decreased growth rate at pH levels below ∼7.5), blue mussel, *Mytilus edulis* (decreased size at pH ∼7.6), spotted wolffish (growth rate decrease at pH ∼6.4), and blue crab (increased calcification rate starting at pH ∼7.8).

### Comparison of species thresholds against environmental changes

**Fig 4:**
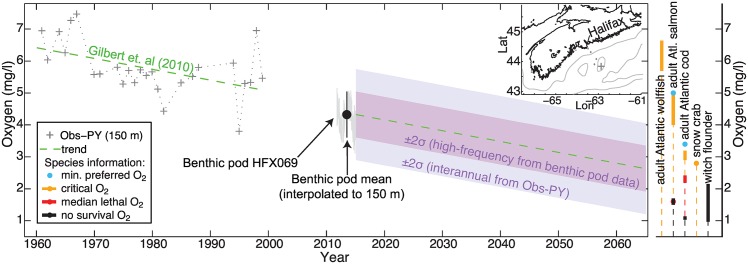
Time series of central Scotian Shelf oxygen observations and trend at 150 m depth (a.) with oxygen information for a species subset (b). (a.) The mean oxygen content (black circle) +/- 2 standard deviations (black vertical bar) for the 2012-2015 observing period from benthic pods HFX098 (133 m) and HFX069 (160 m), interpolated to 150 m, superimposed on the original time series from benthic pod HFX069 (light gray). The oxygen trend of Gilbert et al. [42] (green dashed line) was calculated using the central Scotian Shelf 150 m oxygen anomalies from 1961 to 1999 from Petrie and Yeats [47] (Obs-PY, grey crosses). The locations of Obs-PY and the benthic pods are indicated on the inset map (cross and diamonds, respectively; contour lines = 100 m, 200 m depth). The oxygen trend (green dashed line) is plotted over the next 50 years (until 2065) starting from the benthic pod 150 m mean oxygen value, and we represent variability from short term fluctuations (dark purple shading = +/- 2 standard deviations from benthic pod measurements) and interdecadal changes (light purple shading = +/- 2 standard deviations from the detrended anomalies of Petrie and Yeats [47]). (b.) Oxygen information for a subset of 5 species is plotted for comparison: minimum preferred oxygen (blue), critical oxygen level (orange), median lethal oxygen level (red), no survival oxygen level (black).

Gilbert et al. [42] identified a multidecadal oxygen trend of −1.06 mmol m^−3^ yr^−1^, based on the time series of Petrie and Yeats [47]. Starting from the mean oxygen state at 150 m (estimated from benthic pod data), we compare species information against the hypothetical continuation of the observed oxygen trend over the next half century (Fig 4). The estimated interannual and higher-frequency variability is represented by ±2*σ* where *σ* is the standard deviation of the historical and benthic pod oxygen time series, respectively. Here, we present the historical time series of oxygen anomalies assuming a 5.7 mg/l central value. This central value compares well against both the pre- and post-1980 bottom oxygen climatologies of Bianucci et al. [73] at 150 m depth near the trend location, and a subset of the original historical data (B. Petrie, personal communication, 2016).

The intersection of the species critical oxygen level with the trend predicts the timing of oxygen limitation. Whereas the mean oxygen state at 150 m on the central Scotian Shelf is already below the critical oxygen level of adult Atlantic wolffish, and within the range of critical oxygen estimates of Adult Atlantic salmon, oxygen is not yet limiting for adult Atlantic cod. The critical oxygen level of adult Atlantic cod intersects the trend in year 2047. When variability is considered, adult Atlantic cod could experience a critical oxygen level much earlier: in 2026 based on high-frequency variability, and at present using the estimate of interannual variability. The median lethal oxygen level of Adult Atlantic cod intersects the trend after 2065, but could occur as early as year 2028 based on interannual variability. Witch flounder’s no survival oxygen level intersects the trend including variability in the next 20 to 40 years—in years 2036 and 2057 for interannual and high-frequency variability, respectively (see Fig 4).

**Fig 5:**
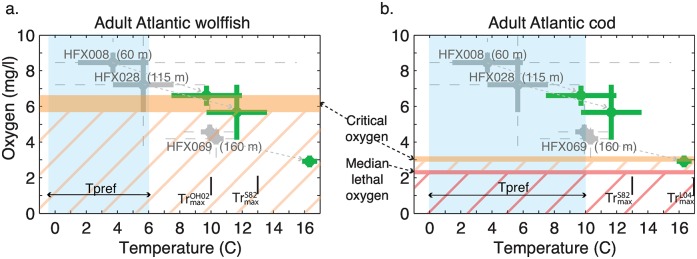
Species thermal and oxygen requirements vs present conditions and future scenarios on the central Scotian Shelf. Benthic pod temperature and oxygen observations from the 2012-2015 observing period are represented by the long term mean values (grey circles), +/- 2 standard deviations (thick grey bars), and the total range of observed values (grey dashed lines). We superimpose the simulated temperature, salinity, and/or oxygen changes from high-resolution modelling experiments for Scenario III ([73, 74]; described in the text and Table 1) to the long term mean values at benthic pods HFX008 (60 m), HFX028 (115 m), and HFX069 (160 m) (green circles). We represent future variability with +/- 2 standard deviations calculated from benthic pod data (thick green bars). The environmental requirements of (a.) adult Atlantic wolffish and (b.) adult Atlantic cod for temperature and oxygen are overlain, including the total range of literature-derived values of the preferred temperature (Tpref = light blue shading) and the upper limit(s) of the animal’s temperature range (Tr_*max*_ = black vertical bar) from all available sources: OH02 = [91]; S82 = [92]; L04 = [93]. The critical oxygen level (orange horizontal bar) and, in the case of adult Atlantic cod, the median lethal oxygen level (red horizontal bar) are shown. Conditions that are sub-optimal with respect to oxygen are indicated by orange (red) diagonal lines to indicate oxygen concentrations below the oxygen critical (median lethal) level. Note that interactions between environmental variables are not considered.

We compare the future conditions at benthic pod locations with the oxygen and temperature information for adult Atlantic wolffish and adult Atlantic cod in Fig 5. Our comparison treats the environmental variables as independent, as we do not consider interactions between temperature and oxygen and the combined effect on the species. Adult Atlantic wolffish encounter unsuitable thermal and oxygen conditions at all benthic pod sites in the future scenario (Fig 5a). Scenario III warming (+6°C) results in temperatures at all benthic pod sites well above the adult Atlantic wolffish’s preferred temperature (by ∼3.5 − 10°C). The future scenario surpasses the species’ reported temperature ranges (upper limits of 10°C [91] and 13°C [92]) at site HFX069 (150 m), and exceeds the O’Dea and Haedrich [91] 10°C value at site HFX028 (115 m). While the future scenario mean temperature at site HFX008 (60 m) is just below 10°C, this threshold is exceeded when variability is considered (e.g. the green bars representing ±2*σ*). Mean oxygen concentrations are at or below the critical oxygen level for adult Atlantic wolffish at all benthic pod sites in the future scenario.

Adult Atlantic cod lose preferred temperature conditions in the future scenario: the mean temperature exceeds 10°C at HFX028 (115 m) and HFX069 (160 m), and the mean temperature at HFX008 (60 m) is 9.73°C, such that variability at this shallow site produces temperatures above the preferred temperature upper limit (Fig 5b). The deepest site, HFX069, becomes unsuitable for adult Atlantic cod, as the future scenario mean value is at the critical oxygen level, several degrees warmer than the maximum thermal limit reported by Scott [92], and within 0.7°C of the maximum thermal limit reported by Lough [93]. The temperature at HFX028 fluctuates above the maximum temperature reported by Scott [92] (based on temperature variability). However, oxygen is not limiting at sites HFX008 and HFX028, and we note the future scenario oxygen remains above the median lethal oxygen level of adult Atlantic cod. Temperature changes are thus more limiting than oxygen changes for adult Atlantic cod in this future scenario on the central Scotian Shelf, with the exception of the deepest benthic pod site (HFX069) where both temperature and oxygen impact species performance.

## Discussion and Conclusions

Combining predictions of future ocean temperature, salinity, oxygen and pH with a set of realistic limits of marine species inhabiting the region is an important long-term goal, and this literature review constitutes a step towards this goal. Temperature and oxygen requirements of important fish and macroinvertebrate species from the Gulf of St. Lawrence and Scotian Shelf, two economically important marine ecoregions in Eastern Canada, are investigated. Oxygen declines and temperature increases are occurring in both regions, and vulnerability to changes in temperature and oxygen differ from one species to the next. Most species in our study region exhibit narrow temperature preferences, and suffer negative impacts (i.e. based on exceeding critical oxygen, median lethal or no survival levels) above the typical hypoxia threshold (2 mg/L) (cf. [94]).

### Limitations and Insights from Literature Review

Marine species’ oxygen preferences and requirements are less well characterized than temperature requirements. Whereas temperature information is found for 68 lifestage-species, fewer oxygen category observations are available (N = 24, 31, 32, and 24 lifestage-species have known preferred, critical, median lethal, and no survival oxygen levels, respectively), and estimates for all four oxygen categories are only found for two lifestage-species, the adult Atlantic salmon and adult Atlantic cod. Data deficiency may produce incomplete habitat predictions (and the juvenile and larval stages are particularly data deficient) (see S1–S5 Tables). Beyond missing individual categories or variables, further experimental work is needed to understand the combined effects of multiple environmental variables (e.g. multi-stressor experiments) on a species-lifestage.

The environmental requirements of some species had been researched multiple times (see S1–S5 Tables). In general, results closely matched one another—multiple studies produced similar temperature ranges for the adult winter flounder, witch flounder and adult Atlantic wolffish, preferred temperatures for adult deep sea scallop, critical oxygen levels for adult Atlantic salmon, and median lethal oxygen levels for juvenile blue crab and juvenile summer flounder. However, conflicting temperature values were found for the winter flounder, red hake, and witch flounder (S2 Table), and different oxygen results were found for the ocean quahog (critical oxygen), molting to post larval Say’s mud crab and American oyster (median lethal oxygen). In the case of the American oyster, the multiple values for median lethal oxygen level are the result of varying experimental temperature and salinity [95], clearly demonstrating the influence of other environmental variables on a species tolerance to low oxygen.

Davis [65] noted that contradictory experimental results for the same organism can make it difficult to establish actual critical oxygen levels. Inconsistencies between critical oxygen definitions were evident during this literature review. However, critical oxygen is a useful manner of summarizing the many physiological and behavioural effects that low oxygen conditions produce in different species and phyla. For a thorough review and analysis of critical oxygen, see the discussion by Rogers et al. [10]. The preferred category is also flawed because it is often based on observations rather than experimental results. Observations based on one population or location may not reflect the entire preferred oxygen range of a particular species.

### Species environmental tolerances in the context of environmental change

We evaluate to what extent a subset of species may be affected by low oxygen over the next half century, by (1) comparing the reported environmental limits against the continuation of an observed long-term oxygen trend on the central Scotian Shelf at 150 m, and (2) presenting one possible future scenario for the central Scotian Shelf bottom waters based on recent benthic pod observations combined with ocean modelling results, and comparing the future scenario temperature and oxygen conditions against the preferred temperature, maximum temperature, and critical and median lethal oxygen levels reported for two vulnerable species, adult Atlantic wolffish and adult Atlantic cod.

The extension of an observed oxygen trend provides a first-order prediction of how changes in environmental conditions may affect regional species. Although the linearly extended trend is unlikely to yield accurate timing of regional species oxygen stress and disappearance, the expected order in which different species may experience changes in habitat is likely robust, which can help direct future research and monitoring. While the mean oxygen state at this location is already below (at) the critical oxygen level of adult Atlantic wolffish (adult Atlantic salmon), even species able to tolerate lower oxygen levels (e.g. adult Atlantic cod, witch flounder) are likely to experience unsuitable oxygen conditions in the next few decades when large natural variability is superimposed on the trend.

The environmental tolerances and thresholds identified from the literature are not independent for a given species. As temperatures increase, metabolic rates rise, increasing oxygen demand, while oxygen solubility decreases. Species sensitivity to low oxygen is thus increased in warmer conditions, and critical oxygen values increase with temperature [96]. Habitat loss will likely occur once critical oxygen levels are reached. We thus expect that future warming will further affect oxygen habitats due to the non-independence of temperature and oxygen effects on species. However, experimental estimates of critical oxygen or median lethal oxygen levels do not sufficiently measure this dependence. For example, Deutsch et al. [20] identified only 16 species for which oxygen thresholds are experimentally determined using at least three temperatures in their survey of the literature. If climate change predictions are to be understood in terms of impacts to fish species, it is imperative that a greater number of experiments be carried out to evaluate temperature and oxygen interactions in fish respiration.

Our comparison of species tolerances to environmental change is limited to an independent consideration of environmental variables, as they are directly reported from the literature. We therefore do not account for potentially important interactions of changes in multiple environmental variables. According to Pörtner [23, 24], subjecting an organism to multiple stressors (e.g. lowering oxygen concentration below an organism’s critical oxygen level and/or lowering pH) is physiologically equivalent to narrowing its window of thermal tolerance. Extending this understanding to the oxygen and temperature thresholds shown in Fig 5, as the oxygen concentration falls below the reported critical oxygen level, the maximum Tr should decrease.

In the experimental oxygen studies included in this literature review, the environmental stress time scale varied between 2 hours and 28 days of exposure (see descriptions in S1 Table). The time scale over which environmental conditions fluctuate and a stressful habitat persists (i.e. elevated temperature, low pH, or low oxygen concentration) may affect the type of organismal response, and result in different impacts. Avoidance is a key behavioral response mobile marine animals employ when confronted with oxygen concentrations associated with impacts to growth [78]. Benthic species that are sessile or have reduced mobility are unable to avoid low oxygen conditions. However, benthic species typically have lower oxygen demands than mobile species, are more tolerant to low oxygen, and have a high capacity for adaptation (e.g. crustaceans are known to adapt to low oxygen conditions by various mechanisms increasing the efficiency of oxygen uptake from the environment) [97].

In the case of short-term exposure to a low oxygen habitat, fish can increase gill ventilation (at an energetic cost) to attempt to maintain oxygenation. When increased gill ventilation cannot compensate for low oxygen concentration, fish can reduce activities such as swimming and feeding, and decrease growth [19]. If the stress continues over the long term, chronic exposure is associated with impacts on fish growth, reproduction, and immune system functioning [98]. Whereas changes in fish hemoglobin concentration and isomorphs have been observed under long-term exposure to low oxygen conditions, the potential for physiological adaptation is reduced by short-term (i.e. fluctuating) oxygen conditions ([98] and references therein).

### Consideration of Adaptation

Species ability to adapt to future environmental conditions remains uncertain. As ambient temperature nears range extremes, energy allocation towards growth and reproduction declines (i.e. increasing temperature produces decreased Atlantic cod size) [99]. Since ocean temperatures are increasing both globally and in this region, a future size shift to smaller, earlier-reproducing individuals is a possibility. Increasing temperatures combined with decreasing oxygen yielded a reduction of 14–24% in maximum body weight in a global model representing 610 demersal fish species for a high emissions scenario by year 2050, relative to year 2000 [100]. However, early life stages may be the most sensitive to the effects of climate change [101], confounding potential growth strategies of future fish populations. Whether the species-specific thresholds compiled for the Scotian Shelf and Gulf of St. Lawrence region would hold in other global regions (e.g. the eastern Atlantic) is of interest, as it may inform our understanding of species’ adaptation capabilities.

It is well documented that marine populations can adjust or adapt to changes in selective pressures, either biotic or abiotic [102]. We acknowledge that the environmental envelopes compiled in our literature review do not account for such adaptive capacity. In particular, experiments employing short term environmental change neglect evolutionary responses [103, 104]. Plastic responses to new selective gradients can manifest in physiological, morphological, or behavioral changes (e.g. as discussed for responses to low pH [104] and low oxygen [97, 105]. Given that numerous phenotypes are possible for a single genotype, some of these phenotypes will be associated with higher fitness in a warmer, lower oxygen, or lower pH environment. Discussions of evolutionary biochemical adaptation including changes in gene expression and protein structure and function to warming [25] and lower pH [26] suggest that marine organisms hold the potential to adapt.

Adaptive capacity is generally supported by traits describing high growth rates, large genetic variability, short generation time, and greater phenotype plasticity [102, 106, 107]. Limits to adaptive capacity can be related to high environmental variability [108], high rates of environmental change, and the extent to which species reside very close to their thermal limit [25, 107]. For the example of cod residing at the southern limit of their distribution (thus, likely close to their thermal limit) and facing high rates of warming, adaptive capacity is likely low. A meta-analysis of the responses of marine life to climate change revealed that approximately one quarter exhibited no response, which could be due to evolutionary adaptation among other possible causes [109].

### Implications for Fisheries

The observed oxygen trend [42] occurred over a time period during which fish stocks declined (e.g., silver hake, Atlantic salmon, and Atlantic wolffish). Fishery declines may not result solely from overfishing but also from changes in environmental conditions (e.g. [110]). Demersal fish (e.g. Atlantic wolffish, Atlantic cod) may be especially vulnerable to habitat loss through the effects of vertical compression from climate change. Wolffish decline has been observed since 1978, and the average size of Atlantic wolffish residing in the southern GSL and SS has decreased by approximately 50% between the mid-1980s and early 2000s [91]. The timing of decreased wolffish abundance and distribution changes on the Scotian Shelf appears to coincide with bottom oxygen declines [73]. In the future scenario, adult Atlantic wolffish encounter unsuitable thermal and oxygen conditions at all central Scotian Shelf benthic pod sites.

Atlantic cod on the Scotian Shelf, where the species resides at the southern limit of its distribution, will likely confront future environmental conditions that exceed its thermal tolerance [111]. Based on a metabolic index combining the effects of temperature increases and oxygen declines, Deutsch et al. [20] predicted significant habitat loss (resulting from vertical habitat compression and a shorter habitable season) for Atlantic cod on the Scotian Shelf and throughout the southernmost areas of the species distribution, by the end of the century. Our results suggest that in the case of the Atlantic cod decline, overfishing was very likely the main cause, given that regional trends do not indicate habitat unsuitability until years afterward. In our future scenario, adult Atlantic cod are more impacted by warming than oxygen declines, except at the deepest site where both temperature and oxygen levels are unfavorable.

In the case of the species requiring the coldest temperatures—snow crab and Greenland halibut, no longterm declines have yet been observed. In fact, these species are currently abundant in the Scotian Shelf and Gulf of St. Lawrence region, with the snow crab ranking second in both commercial shellfish landings and value for the region, and halibut ranking third in landings and first in value for commercial fin fish [8]. Notably, the southern extent of these species’ ranges is the eastern and western Scotian Shelf for snow crab and Greenland halibut, respectively [112, 113]. In a 50-year prediction (the decade 2060-2070), Chabot et al. [12] concluded that warming of shelf and slope bottom waters would lead to commercial extinction of snow crab in the eastern SS and St. Lawrence Estuary, while Greenland halibut could potentially be lost from the Gulf of St. Lawrence, with warmer waters negatively affecting productivity, abundance, and distribution.

Future fishing strategies will need to consider not only individual fish stocks, but also changing climate-driven habitat variables alongside species requirements. Regional fisheries management must be informed by an integrated ecosystem assessment [114] considering climate change impacts, cumulative impacts, and complex interactions (e.g. [115]). For example, warming ocean temperatures are predicted to cause species losses on the Scotian Shelf, although species gains from lower latitudes are also predicted for most Canadian waters [7]. Whether fisheries can effectively adapt to changes in species distributions is an open question [116].

If regional warming and deoxygenation continue, many species will encounter unsuitable temperature or oxygen conditions within the next century. Projected regional temperature trends and natural variability are both large, and natural variability will act to alternately amplify and dampen anthropogenic warming. When combined with predictions from biogeochemical models, this dataset can aid in the prediction of habitat loss and species shifts under the complex and dynamic ocean conditions in the Gulf of St. Lawrence and Scotian Shelf.

## Supporting Information

S1 Table Oxygen levels of Scotian Shelf and Gulf of St. Lawrence marine species identified from literature survey. Each row reports data for one species, life stage (if available), and source reference. Latin name, common name, and habitat zone (pelagic, demersal, benthic, infaunal) are given with the original reported oxygen values, followed by the oxygen categories (converted to mg/l) of minimim and maximum preferred oxygen, critical oxygen, median lethal (LC50), and lethal (no survival). The data type is described as experimental or observational, with notes indicating experimental conditions, species impacts, and salinity data selected from the World Ocean Database for unit conversion. Oxygen concentrations are converted into units of mg/L, one of the most common oxygen units in physical and environmental studies (Ekau et al., 2010) using unit conversions adapted from the literature and online databases (below). When original units are given as percent saturation, salinity, temperature, and pressure are required to convert to mg/L. If the original study provided such detail, we use the US Geological Survey online conversion (2013). Sea surface pressure is assumed when pressure information is unavailable. While experimental temperature information is always included, salinity information is not described in several experimental studies. In those cases, we accessed surface salinity data (top 2 m) from the World Ocean Database (WOD) (NODC, 2011) collected nearest the experimental date and location coordinates. If the exact location is not given, we estimated the coordinates at which the experiment occurred.
(XLS)

S2 Table Temperature levels of Scotian Shelf and Gulf of St. Lawrence marine species identified from literature survey. Each row reports data for one species, life stage (if available), and source reference. Latin name, common name, and habitat zone (pelagic, demersal, benthic, infaunal) are given, followed by the temperature categories of minimim and maximum temperature, preferred temperature, intolerant temperature, and lethal temperature. The notes indicate specific location and species impacts, if available.
(XLS)

S3 Table Salinity levels of Scotian Shelf and Gulf of St. Lawrence marine species identified from literature survey. Each row reports data for one species, life stage (if available), and source reference. Latin name and common name are given, followed by the categories of minimim and maximum salinity range, preferred salinity, intolerant salinity, and lethal salinity. The notes indicate specific location and species impacts, if available.
(XLS)

S4 Table Depth levels of Scotian Shelf and Gulf of St. Lawrence marine species identified from literature survey. Each row reports data for one species, life stage (if available), and source reference. Latin name, common name, and habitat zone (pelagic, demersal, benthic, infaunal) are given, followed by the depth categories of minimum and maximum depth, mean depth, and minimum and maximum preferred depth. The notes indicate specific location and species impacts, if available. Sources are listed for the depths and the location. Locations are listed as Scotian Shelf (SS) or Gulf of St. Lawrence (GSL).
(XLS)

S5 Table pH information for Scotian Shelf and Gulf of St. Lawrence marine species identified from literature survey. Each row reports data for one species, life stage (if available), and source reference. Habitat zone (pelagic, demersal, benthic, infaunal) is given. The data description specifies the information as experimental (Exp.) or observational (Obs.) and the measured variable. The source reference is given, along with the reference code used in Fig 3. The evaluated pH levels are indicated (from high to low pH), and the respective measured response is coded as 0 = ‘no impact’, 1 = ‘impact is a decrease’, or 2 = ‘impact is an increase’. Notes on experimental outcomes are provided.
(XLS)

S1 Text References cited in Supplemental Tables. (DOC)
