# Supplementary material for: Putting Temperature and Oxygen Thresholds of Marine Animals in Context of Environmental Change: A Regional Perspective for the Scotian Shelf and Gulf of St. Lawrence
Source: PLoS One. 2016 Dec 20;11(12):e0167411. doi: 10.1371/journal.pone.0167411 (PMC5172530; doi:10.1371/journal.pone.0167411)
Supplement: S1 Text — (DOC) [file pone.0167411.s006.doc]

**S1 Text. Supplement References**

Able, K. W., and Fahay, M. P. 2010. Ecology of estuarine fishes*: temperate waters of the western north Atlantic*. Baltimore: The Johns Hopkins University Press.

Able, K. W., Balletto, J. H., Hagan, S. M., Jivoff, P. R., and Strait, K. 2007. Linkages between salt marshes and other nekton habitats in Delaware Bay, USA. Rev. Fish. Sci. **15**:1-61.

Abraham, B. J. 1985. Species Profiles: life histories and environmental requirements of coastal fishes and invertebrates (Mid-Atlantic) – mummichog and striped killifish. U.S. Fish Wildl. Serv. Biol. Rep. 82(11.40). U.S. Army Corps of Engineers. 23 pp.

Ahrenholz, D. W., Guthrie, J. F., and Krouse, C. W. 1989. Results of abundance surveys of juvenile Atlantic and Gulf menhaden, *Brevoortia tyrannus* and *B. patronus*. NOAA Tech. Rep. NMFS 84. 14 pp.

Allen, J. A. 1959. On the biology of *Pandalus borealis* (Krøyer), with reference to a population off the Northumberland coast. Journal of the Marine Biological Association of the United Kingdom **38**(1), 189-220, doi:10.1017/S002531540001568X.

Altman, I. 2010. *Trematode parasites of the mudsnail ilyanassa obsoleta: an analysis of parasite communities at different scales*. Doctoral dissertation, University of New Hampshire.

Anderson, G. 1985. *Species Profiles: life Histories and environmental requirements of coastal fishes and invertebrates (Gulf of Mexico) -- grass shrimp*. U.S. Fish Wildl. Serv. Biol. Rep. 82(11.35). U.S. Army Corps of Engineers: TR EL-82-4. 19 pp.

Annis, E. R. 2005. Temperature effects on the vertical distribution of lobster postlarvae (Homarus americanus). Limnol. Oceanogr. **50**(6): 1972-1982.

Arendt, M. D., Lucy, J. A., and Evans, D. A. 2001. Diel and seasonal activity patterns of adult tautog, *Tautoga onitus* (Labridae), at manmade and natural structures in lower Chesapeake Bay. Fish. Bull. **99**: 519-527.

Arnberg, M., Calosi, P., Spicer, J.I., Tandberg, A.H.S., Nilsen, M., Westerlund, S. and Bechmann, R.K. 2012. Elevated temperature elicits greater effects than decreased pH on the development, feeding and metabolism of northern shrimp (*Pandalus borealis*) larvae. Mar. Biol., **160**(8): 2037-2048.

Atlantic Wolffish Biological Review Team. 2009. Status review of Atlantic wolffish (*Anarhichas lupus*). Report to National Marine Fisheries Service, Northeast Regional Office. September 30, 2009.

Barbeau, M.A., and R.E. Schleibling. 1994. Temperature effects on predation of juvenile sea scallops Placopecten magellanicus (Gmelin) by sea stars (Asterias vulgaris Verrill) and crabs (Cancer irroratus Say). Journal of Experimental Marine Biology and Ecology **182**: 27-47.

Bechmann, R. K., Taban, I. C., Westerlund, S., Godal, B. F., Arnberg, M., Vingen, S., Ingvarsdottir, A. and Baussant, T. 2011. Effects of ocean acidification on early life stages of shrimp (Pandalus borealis) and mussel (Mytilus edulis). *J. Toxic. Envir. Health A*, *74*(7-9), 424-438.

Bejda, A. J., Studholme, A. L., and Olla, B. L. 1987. Behavioral responses of red hake, Urophycis chuss, to decreasing concentrations of dissolved oxygen. *Environmental Biology of Fishes*, ***19***(4): 261-268.

Bejda, A. J., Phelan, B. A., and Studholme, A. L. 1992. The effect of dissolved oxygen on the growth of young-of-the-year winter flounder, Pseudopleuronectes americanus. *Environmental Biology of Fishes*, ***34***(3): 321-321.

Berge, J. A., Bjerkeng, B., Pettersen, O., Schaanning, M. T., and Øxnevad, S. 2006. Effects of increased sea water concentrations of CO2 on growth of the bivalve *Mytilus edulis* L. Chemosphere, *62*(4), 681-687.

Bergström, B.I. 2000. The biology of *Pandalus*. Advances in Marine Biology, **38**, 55-245.

Bigelow, H. B., and Schroeder, W. C. 1953. *Fishes of the Gulf of Maine,* 53. Washington, DC: US Government Printing Office.

Bigford, T. E. 1979. Synopsis of biological data on the rock crab, *Cancer irroratus Say*. NOAA Technical Report NMFS Circular 426. *FAO Fisheries Synopsis*, 123.

Bogdanov, A.S., S.I. Doroshev, and A.F. Karpevich. 1967. Experimental transfer of *Salmo gairdneri* and *Roccus saxatilis* from the USA for acclimatization in bodies of water of the USSR. Voprosy Ikhtiologii 42: 185-187. (Translated from Russian by R. M. Howland, Narragensett Marine Game Fish Research Laboratory, Rhode Island.)

Bowering, W. R. 1993. Underwater world: Turbot (Greenland halibut). Available from the Communications Directorate, Department of Fisheries and Oceans, Ottawa, Ontario. DFO/4665. UW/24.

Bowering, W. R., and Nedreaas, K. H. 2000. A comparison of Greenland halibut (*Reinhardtius hippoglossoides* (Walbaum)) fisheries and distribution in the Northwest and Northeast Atlantic. Sarsia, **85**(1): 61-76.

Bowman, R. E., Stillwell, C. E., Michaels, W. L., and Grosslein, M. D. 2000. Food of Northwest Atlantic fishes and two common species of squid. NOAA Technical Memorandum NMFS-NE-155. U.S. Department of Commerce, Washington, D.C.

Boyar, H. C. 1964. Occurrence of the Greenland halibut, Reinhardtius hippoglossoides (Walbaum), in shallow waters in the Gulf of Maine. *Copeia*, **1**: 232-233.

Breen, E., and Metaxas, A. 2009. Effects of juvenile non-indigenous *Carcinus maenas* on the growth and condition of juvenile *Cancer irroratus*. *Journal of experimental marine biology and ecology*, ***377***(1): 12-19.

Breitburg, D. L. 1994. Behavioral response of fish larvae to low dissolved oxygen concentrations in a stratified water column. Mar. Biol. **120**(4): 615–625.

Buckley, J. 1989. Species profiles: life histories and environmental requirements of coastal fishes and invertebrates (North Atlantic) – winter flounder. U.S. Fish Wildl. Serv. Biol. Rep. 82(11.87). U.S. Army Corps of Engineers, TR EL-82-4. 12 pp.

Burton, D. T., Richardson, L. B., and Moore, C. J. 1980. Effect of oxygen reduction rate and constant low dissolved oxygen concentrations on two estuarine fish. *Transactions of the American Fisheries Society*, ***109***(5): 552-557.

Butler, P.A. 1954. Summary of our knowledge of the oyster in the Gulf of Mexico. Fish. Bull., U.S. Fish and Wildl. Serv. 55(89): 479-489.

Cairns, D. K. 2001. An evaluation of possible causes of the decline in pre-fishery abundance of North American Atlantic salmon. Can. Tech. Rep. Fish. Aquat. Sci. No. 2358.

Capossela, K. M. 2010. *Migration dynamics, within-estuary behaviors and cardiorespiratory responses of summer flounder to selected estuarine conditions.* Doctoral dissertation, The College of William and Mary.

Cargnelli, L. M., Grisebach, S. J., Packer, D. B., Berrien, P. L., Morse, W. W., and Johnson, D. L. 1999. Essential fish habitat source document: 
Witch flounder, *Glyptocephalus cynoglossus*, life history and habitat characteristics. NOAA Technical Memorandum NMFS-NE-139. U.S. Department of Commerce, Washington, D.C.

Castagna, M., and Chanley, P. 1973. Salinity tolerance of some marine bivalves from inshore and estuarine environments in Virginia waters on the western mid-Atlantic coast. *Malacologia*, ***12***(1): 47-96.

Chabot, D. and Gilbert, D. 2013. Description of the Atlantic Large Aquatic Basin as a habitat for marine fish and invertebrates. Ch. 1 (p. 1-15) *In*: Climate change impacts, vulnerabilities and opportunities analysis of the Marine Atlantic Basin (Shackell, N.L, B.J.W. Greenan, P. Pepin, and A. Warburton, eds.), Can. Manuscr. Rep. Fish. Aquat. Sci. **3012**: xvi+355 p.

Chabot, D., Guénette, S. and Stortini, C. 2013. A review of the physiological susceptibility of commercial species of fish and crustaceans of the northwest Atlantic to changes in water temperature, dissolved oxygen, pH and salinity. Ch. 4 (p. 83-167) *In*: Climate Change Impacts, Vulnerabilities and Opportunities Analysis of the Marine Atlantic Basin [Shackell, N.L, Greenan, B.J.W., Pepin, P., Chabot, D. and Warburton, A. (Eds.)]. Can. Manuscr. Rep. Fish. Aquat. Sci. 3012: xvi+355 p.

Chakraborty, R. 1993. Analysis of genetic structure of populations: Meaning, methods, and implications. *In* Human population genetics. *Edited by* P. P. Majumder. Springer U.S., New York, N.Y. pp. 189–206.

Chang, S., Berrien, P. L., Johnson, D. L. and Morse, W. W. 1999. Essential Fish Habitat Source Document:Windowpane, *Scophthalmus aquosus*, Life History and Habitat Characteristics. NOAA Technical Memorandum NMFS-NE Ser. Woods Hole, M.A.

Chaput, G. and Bradford, R. G. 2003. American shad (Alosa sapidissima) in Atlantic Canada. Can. Sci. Advis. Sec. Res. Doc. 2003/009. 71 pp.

Charmantier, G., Charmantier-Daures, M., Bouaricha, N., Thuet, P., Aiken, D. E. and Trilles, J.-P. 1988. Ontogeny of osmoregulation and salinity tolerance in two decapod crustaceans: *Homarus americanus* and *Penaeus japonicus*. *Biol. Bull.* **175**: 102–110.

Charmantier, G., Haond, C., Lignot, J.-H., and Charmantier-Daures, M. 2001. Ecophysiological adaptation to salinity throughout a life cycle: a review in homarid lobsters. *Journal of Experimental Biology*, ***204***(5), 967-977.

Chittenden, M. E., Jr. 1969. Life history and ecology of the American shad, *Alosa sapidissima*, in the Delaware River. Doctoral dissertation, Rutgers University. 458 pp.

Cohen, D. M., Inada, T., Iwamoto, T., and Scialabba, N. 1990. Gadiform fishes of the world (Order Gadiformes). An annotated and illustrated catalogue of cods, hakes, grenadiers and other gadiform fishes known to data. *FAO Fisheries Synopsis*, **10**(125), 452.

Collette, B.B., and Klein-MacPhee, G. 2002. Bigelow and Schroeder’s fishes of the Gulf of Maine. 3rd ed. Smithsonian Institution Press, Washington, D.C.

Collins, M. A. J. 1978. Experiments on the hatching period of the eggs of the lumpfish *Cyclopterus lumpus L.* in *Newfoundland waters. Naturaliste Can.*, *105*: 169-171.

Condron, A., DeConto, R., and Friedland, K. 2002. Paleoclimate variability in ocean conditions and the production of North American Atlantic salmon. NPAFC Technical Report No. 4.

Corey, S. 1981. The life history of *Crangon septemspinosa Say* (Decapoda, Caridea) in the shallow sublittoral area of Passamaquoddy Bay, New Brunswick, Canada. *Crustaceana*, 41(1): 21-28.

COSEWIC. 2011. COSEWIC assessment and status report on the Atlantic salmon, Salmo salar (Nunavik population, Labrador population, northeast Newfoundland population, south Newfoundland population, southwest Newfoundland population, northwest Newfoundland population, Québec eastern north shore population, Québec western north shore population, Anticosti Island population, inner St. Lawrence population, Lake Ontario population, Gaspé-southern Gulf of St. Lawrence population, eastern Cape Breton population, Nova Scotia southern upland population, inner Bay of Fundy population, outer Bay of Fundy population) in Canada. [Online]. Committee on the Status of Endangered Wildlife in Canada, Ottawa, O.N. Available from http://www.sararegistry.gc.ca/virtual_sara/files/cosewic/sr_Atlantic%20Salmon_2011_e.pdf [accessed 22 July 2013].

Covich, A. P., Austen, M. C., Barlocher, F., Chauvet, E., Cardinale, B. J., Biles, C. L., and Moss, B. 2004. The role of biodiversity in the functioning of freshwater and marine benthic ecosystems. Bioscience, **54**(8): 767–775.

Cox, P., and Anderson, M. 1922. A study of the lumpfish (*Cyclopterus lumpus L.*). *Contributions to Canadian Biology and Fisheries*, **1**(1): 1-20.

Cranford, P. J. 1988. Behaviour and ecological importance of a mud snail (*Ilyanassa obsoleta*) population in a temperate macrotidal estuary. Can. J. of Zoolog. **66**(2): 459–466.

Cross, J. N., Zetlin, C. A., Berrien, P. L., Johnson, D. L., and McBride, C. 1999. Essential Fish Habitat Source Document: Butterfish, *Peprilus triacanthus*, life history and habitat characteristics. NOAA Tech. Mem. NMFS-NE-145. Woods Hole, M.A. 42 pp.

D’Amours, D. 1993. The distribution of cod (*Gadus morhua*) in relation to temperature and oxygen level in the Gulf of St. Lawrence. *Fish. Oceanogr.* **2**(1): 24-29.

Davenport, J. 1983. Oxygen and the developing eggs and larvae of the lumpfish, *Cyclopterus lumpus*. J. Mar. Biol. Assoc. U.K., **63**(03): 633-640.

Dawe, E. G., Walsh, S. J., and Hynick, E. M. 2010. Capture efficiency of a multi-species survey trawl for Snow Crab (*Chionoecetes opilio*) in the Newfoundland region. *Fish. Res.*, **101**(1): 70-79.

Davis, J. R., and Cheek, R. P. 1966. Distribution, food habits, and growth of young clupeids, Cape Fear River system, North Carolina. North Carolina Wildlife Resources Commission. D. J. Rep. F-16-R North Carolina. 18 pp.

Dawe, J. L., and Neis, B. 2012. Species at risk in Canada: Lessons learned from the listing of three species of wolffish. Mar. Policy, **36**(2): 405–413. doi:10.1016/j.marpol.2011.06.010.

DeCola, J. N. 1970. Water quality requirements for Atlantic salmon. No. PB--230733; CWT 10-16. Federal Water Quality Administration, Needham Heights, M.A. 42 pp.

Delbeek, J. C., and Williams, D. D. 1987. Food resource partitioning between sympatric populations of brackishwater sticklebacks. J. Anim. Ecol. 56: 949-967.

De Silva, S. S., and Soto, D. 2009. Climate change and aquaculture: Potential impacts, adaptation and mitigation. *In* Climate change implications for fisheries and aquaculture: Overview of current scientific knowledge. *Edited by* K. Cochrane, C. De Young, D. Soto and T. Bahri. FAO fisheries and aquaculture technical paper No. 530. FAO, Rome, Italy. pp. 151–212.

De Silva, C. D., and Tytler, P. 1973. The influence of reduced environmental oxygen on the metabolism and survival of herring and plaice larvae. *Netherlands J. Sea Res.*, **7**: 345-362.

DFO. 2012a. Variables and units used in the ODMS [online]. Available from http://slgo.ca/app-sgdo/en/docs_reference/variables-unites.html [accessed 29 May 2013].

DFO. 2012b. Temperature threshold to define management strategies for Atlantic salmon (Salmo salar) fisheries under environmentally stressful conditions. DFO Can. Sci. Advis. Sec. Sci. Advis. Rep. 2012/019.

DFO. 2013a. Atlantic zone monitoring program website [online]. Available from http://www.meds-sdmm.dfo-mpo.gc.ca/isdm-gdsi/azmp-pmza/index-eng.html [accessed 16 May 2013].

DFO. 2013b. Canada's fish and seafood industry [online]. Available from http://www.ats-sea.agr.gc.ca/sea-mer/ind-eng.htm [accessed 26 August 2013].

DFO and MNRF. 2008. Conservation status report, Atlantic salmon in Atlantic Canada and Québec: Part I – species information. Can. MS Rep. Fish. Aquat. Sci. No. 2861. 208 pp.

DFO and MNRF. 2009. Conservation status report, Atlantic salmon in Atlantic Canada and Québec: Part II – anthropogenic considerations. Can. Manuscr. Rep. Fish. Aquat. Sci. No. 2870.

Diaz, R. J., and Rosenberg, R. 1995. Marine benthic hypoxia: a review of its ecological effects and the behavioural responses of benthic macrofauna. *Oceanogr. Mar. Biol. Ann. Rev.* **33**: 245-03.

Dimichele, L., and Taylor, M. H. 1980. The environmental control of hatching in Fundulus heteroclitus. *J. Experimen. Zoo.*, **214**(2): 181-187.

Dupont-Prinet, A., Vagner, M., Chabot, D., and Audet, C. 2013. Impact of hypoxia on the metabolism of Greenland halibut (*Reinhardtius hippoglossoides*). Can. J. Fish. Aquat. Sci. **70**(3): 461-469. doi: 10.1139/cjfas-2012-0327.

Factor, J. R. (Ed.). 1995. *Biology of the Lobster: Homarus americanus*. Academic Press, New York.

Fay, C. W., Neves, R. J., and Pardue, G. B. 1983. Species profiles: life histories and environmental requirements of coastal fishes and invertebrates (Mid-Atlantic) – striped bass. U.S. Fish Wildl. Serv., FWS/0BS-82/11.8. U.S. Army Corps of Engineers, TR EL-82-4. 36 pp.

Foss, A., Evensen, T. H., Imsland, A. K., and Øiestad, V. 2001. Effects of reduced salinities on growth, food conversion efficiency and osmoregulatory status in the spotted wolffish. *J. Fish Biol.*, **59**(2): 416-426.

Foss, A., Evensen, T. H., and Øiestad, V. 2002. Effects of hypoxia and hyperoxia on growth and food conversion efficiency in the spotted wolffish  *Anarhichas minor*(Olafsen). Aquacult. Res. **33**(6): 437–444.

Foss A., Røsnes B.A. and Øiestad V. 2003. Graded environmental hypercapnia in juvenile spotted wolffish (*Anarhichas mino*r Olafsen): effects on growth, food conversion efficiency and nephrocalcinosis. Aquaculture, 220(1-4): 607-617.

Foss, A., Kristensen, T., Åtland, Hustveit, H., Hovland, H., Øfsti, A. and Imsland, A.K. 2006. Effects of water reuse and stocking density on water quality, blood physiology and growth rate of juvenile cod (*Gadus morhua*). Aquaculture, 256(1): 255-263.

Fowler, A. E., T. Forsström, M. von Numers and O. Vesakoski. 2013. The North American mud crab Rhithropanopeus harrisii (Gould, 1841) in newly colonized Northern Baltic Sea: distribution and ecology. Aquatic Invasions, 8, 1, 89–96, doi: http://dx.doi.org/10.3391/ai.2013.8.1.10.

Friedland, K. D., Chaput, G., and MacLean, J. C. 2005. The emerging role of climate in post-smolt growth of Atlantic salmon. ICES J. Mar. Sci. **62**(7): 1338–1349. doi:10.1016/j.icesjms.2005.04.013.

Friedland, K. D., Reddin, D. G., and Castonguay, M. 2003. Ocean thermal conditions in the post-smolt nursery of North American Atlantic salmon. ICES J. Mar. Sci. **60**(2): 343–355. doi:10.1016/S1054–3139(03)00022-5.

Frommel, A. Y., Schubert, A., Piatkowski, U., and Clemmesen, C. 2013. Egg and early larval stages of Baltic cod, *Gadus morhua*, are robust to high levels of ocean acidification. *Marine biology*, **160**(8), 1825-1834.

Garcia, E. G., 2007. The northern shrimp (*Pandalus borealis*) offshore fishery in the Northeast Atlantic. Adv. Mar. Biol., **52**: 147-266.

Geshelin, Y., Sheng, J., and Greatbatch, R. J. 1999. Monthly mean climatologies of temperatures and salinity in the western North Atlantic. Can. Tech. Rep. Hydrogr. Ocean Sci. No. 153.

Gibson, R. G. J. 1993. The Atlantic salmon in freshwater: Spawning, rearing and production. Rev. Fish Biol. and Fisher. **3**(1): 39–73.

Goodman, L. R., and Campbell, J. G. 2007. Lethal levels of hypoxia for gulf coast estuarine animals. *Marine Biology*, **152**(1): 37-42.

Gray, C.L. 1992. Tautog *Tautoga onitis*: species profile. Wakefield, RI: Rhode Island Marine Fisheries Section; 49 pp.

Green, J. M., and Farwell, M. 1971. Winter habits of the cunner, *Tautogolabrus adspersus* (Walbaum 1792), in Newfoundland. Can. J. Zoo. **49**(12): 1497-1499.

Gregory, R. S., and Daborn, G. R. 1982. Notes on adult lumpfish *Cyclopterus lumpus* L. from the Bay of Fundy. Proc. N.S. Inst. Sci. **32**: 321-326.

Grosslein, M. D., and Azarovitz, T. R. 1982. *Fish distribution.* MESA New York Bight Atlas Monograph 15. New York Sea Grant Institute, New York. 182 pp.

Grove, M., and Breitburg, D. L. 2005. Growth and reproduction of gelatinous zooplankton exposed to low dissolved oxygen. *Mar. Ecol. Progr. Ser.* **301**: 185-198.

Gutherz, E. J. 1967. Field guide to the flatfishes of the family Bothidae in the western North Atlantic. Fish and Wildlife Service. U.S. Department of the Interior. Circ. 263. 47 pp.

Hales, L. S., Jr., and Able, K. 2001. Winter mortality, growth, and behavior of young-of-the-year of four coastal fishes in New Jersey (USA) waters. *Mar. Biol.* **139**(1), 45-54.

Hall, F. G. 1930. The ability of the common mackerel and other marine fish to remove dissolved oxygen from sea water. *Am. J. Physiol*. **93**(1930): 417-421.

Hamer, H. H., Malzahn, A. M., and Boersma, M. 2011. The invasive ctenophore *Mnemiopsis leidyi*: A threat to fish recruitment in the North Sea? J. Plankton Res. **33**(1): 137–144. doi:10.1093/plankt/fbq100.

Hamwi, A., and Haskin, H. H. 1969. Oxygen consumption and pumping rates in the hard clam Mercenaria mercenaria: a direct method. *Sci.* **163**(3869), 823-824.

Handeland, S. O., Imsland, A. K., and Stefansson, S. O. 2008. The effect of temperature and fish size on growth, feed intake, food conversion efficiency and stomach evacuation rate of Atlantic salmon post-smolts. Aquaculture, **238**(1-4): 36–42. doi:10.1016/j.aquaculture.2008.06.042.

Hansen, L. P., and Quinn, T. P. 1998. The marine phase of the Atlantic salmon (*Salmo salar*) life cycle, with comparisons to pacific salmon. Can. J. Fish. Aquat. Sci. **55**(Suppl. 1): 104–118. doi:10.1139/d98-010.

Hansen, T. K., and Falk‐Petersen, I. B. 2002. Growth and survival of first‐feeding spotted wolffish (*Anarhichas minor* Olafsen) at various temperature regimes. *Aquacult. Res*, **33**(14): 1119-1127.

Harding, G. C. 1992. American lobster (*Homarus americanus* Milne Edwards): A discussion paper on their environmental requirements and known anthropogenic effects on their populations. Can. Tech. Rep. Fish. Aquat. Sci. 1887, 17 pp.

Harding, G. C., Pringle, J. D., Vass, W. P., Pearre Jr., S., and Smith, S. J. 1987. Vertical distribution and daily movements of larval lobsters *Homarus americanus* over Browns Bank, Nova Scotia. Mar. Ecol. Prog. Ser. **41**: 29-41.

Hardy, J. D., Jr. 1978. Development of fishes of the Mid-Atlantic Bight – an atlas of egg, larval, and juvenile stages, vol. II: Anguillidae through Syngnathidae. U.S. Fish and Wild. Serv., U.S. Dep. Interior, FWS/OBS-78/12. 458 pp.

Hardy, J. D., Jr. 1978b. Development of fishes of the Mid-Atlantic Bight – an atlas of egg, larval, and juvenile stages, vol. III: Aphredoderidae through Rachycentridae. U.S. Fish and Wild. Serv., U.S. Dep. Interior, FWS/OBS-78/12. 394 pp.

Harley, C. D. G., Hughes, A. R., Hultgren, K. M., Miner, B. G., Sorte, C. J. B., Thornber, C. S., Williams, S. L. 2006. The impacts of climate change in coastal marine systems. Ecol. Lett. **9**(2): 228–241. doi:10.1111/j.1461-0248.2005.00871.x.

Hart, D. R., and Chute, A. S. 2004. Essential fish habitat source document. Sea scallop, *Placopecten magellanicus*, life history and habitat characteristics. NOAA Tech. Mem. NMFS-NE-189. 21 pp.

Hatin, D., Munro, J., Caron, F., and Simons, R. D. 2007. Movements, home range size, and habitat use and selection of early juvenile Atlantic sturgeon in the St. Lawrence estuarine transition zone. In *American Fisheries Society Symposium.*  **56**:129-155. American Fisheries Society.

Hendrickson, L. 2006. Windowpane Flounder. In Status of the Fishery Resources of the Northeastern United States. Northeast Fisheries Science Center. 13 p. Online: http://www.nefsc.noaa.gov/sos/spsyn/fldrs/window/.

Hendrickson, L. 2008. Gulf of Maine/Georges Bank windowpane flounder: In Northeast Fisheries Science Center. 2008. Assessment of 19 Northeast Groundfish Stocks through 2007: Report of the 3rd Groundfish Assessment Review Meeting (GARM III), Northeast Fisheries Science Center, Woods Hole, Massachusetts, August 4-8, 2008. US Dep Commer, NOAA Fisheries, Northeast Fisheries Science Center Reference Document 08-15. 884 p + xvii.

Herrick, F. H. 1895. The American lobster: A study of its habits and development. Bull. U.S. Fish. Comm. **15**: 1-252.

Holland, J. S., Jr. 1971. Effects of temperature and salinity on growth, food conversion, survival and temperature resistance of juvenile blue crabs, *Callinectes sapidus* Rathbun. 167 pp.

Howell, P. and Simpson, D. 1994. Abundance of marine resources in relation to dissolved oxygen in Long Island Sound. *Estuar*. **17**(2): 394-402.

Imsland, A. K., Foss, A., Sparboe, L. O., and Sigurdsson, S. 2006. The effect of temperature and fish size on growth and feed efficiency ratio of juvenile spotted wolffish *Anarhichas minor*. *J. Fish Biol.* **68**(4): 1107-1122.

Joaquim, N. and Gamperl, A. K. Cardiac function in Atlantic wolffish (*Anarhichas lupus*) explosed to acute temperature and hypoxia challenges. Extended abstract (unpublished data).

Jones, P. W., Martin, F. D., and Hardy, J. D., Jr. 1978. Development of fishes of the Mid-Atlantic Bight: an atlas of egg, larval and juvenile stages, vol. I: Acipenseridae through Ictaluridae. Fish and Wildlife Service, U.S. Department of Interior FWS/OBS-78/12. 366 pp.

Jones, M. 2004. Cultured aquatic species information programme. *Salmo salar* [online]. Available from http://www.fao.org/fishery/culturedspecies/Salmo_salar/en [accessed 26 July 2013].

Jonsson, B., and Jonsson, N. 2009. A review of the likely effects of climate change on anadromous Atlantic salmon *Salmo salar*and brown trout *Salmo trutta*, with particular reference to water temperature and flow. J. Fish Biol. **75**(10): 2381–2447. doi:10.1111/j.1095-8649.2009.02380.x.

Kazakov, R. V., and Khalyapina, L. M. 1981. Oxygen consumption of adult Atlantic salmon (*Salmo salar* L.) males and females in fish culture. *Aquacult.* **25**(2): 289-292.

Keats, D. W., South, G. R., and Steele, D. H. 1985. Reproduction and egg guarding by Atlantic wolffish (*Anarhichas lupus*: Anarhichidae) and ocean pout (*Macrozoarces americanus*: Zoarcidae) in Newfoundland waters. Can. J. Zool. **63**(11): 2565–2568.

Keats, D. W., Steele, D. H., and South, G. R. 1986. Atlantic wolffish (*Anarhichas lupus* L.; Pisces: Anarhichidae) predation on green sea urchins (*Strongylocentrotus droebachiensis* (OF Mull.); Echinodermata: Echinoidea) in eastern Newfoundland. *Can. J. Zool.* **64**(9): 1920-1925.

Keppel, E.A., Scrosati, R.A. and Courtenay, S.C. 2012. Ocean acidification decreases growth and development in American lobster (*Homarus americanu*s) larvae. J. Northw. Atl. Fish. Sci., **44**: 61-66.

King, M. C., and Beazley, K. F. 2005. Selecting focal species for marine protected area network planning in the Scotia–Fundy region of Atlantic Canada. Aquat. Conserv: Mar. Freshw. Ecosyst. **15**(4): 367–385. doi:10.1002/aqc.682.

Kjörsvik, E., Davenport, J. and Lönning, S. 1984. Osmotic changes during the development of eggs and larvae of the lumpsucker, *Cyclopterus lumpus* L. J. Fish Biol. **24**: 311-321.

Klassen, G., and Locke, A. 2007. A biological synopsis of the European green crab, *Carcinus maenas*. Can. Manuscr. Rep. Fish. Aquat. Sci. No. 2818.

Kremer, P. 1994. Patterns of abundance for *Mnemiopsis* in US coastal waters: a comparative overview. *ICES Journal of Marine Science: Journal du Conseil*, **51**(4): 347-354.

Kroglund, F., and Finstad, B. 2003. Low concentrations of inorganic monomeric aluminum impair physiological status and marine survival of Atlantic salmon. Aquaculture, **222**(1–4): 119–133. doi:10.1016/S0044-8486(03)00106-6.

Kulka, D., Hood, C., and Huntington, J. 2007. Recovery strategy for northern wolffish (*Anarhichas denticulatus*) and spotted wolffish (*Anarhichas minor*), and management plan for Atlantic wolffish (*Anarhichas lupus*) in Canada. Fisheries and Oceans Canada, Newfoundland and Labrador Region, St. John’s. N.L.

Kutty, M. N. 1987. Site selection for aquaculture: Chemical features of water. Project Report No. 12(9). African Regional Aquaculture Centre, Port Harcourt, Nigeria.

Larocque, R., Gendron, M. -H., and Dutil, J. D. 2008. A survey of wolffish (*Anarhichas* spp.) and wolffish habitat in Les Méchins, Québec. Can. Tech. Rep. Fish. Aquat. Sci. No. 2786.

Le François, N. R., Desjardins, M., and Blier, P. U. 2004. Enhancement of profitability perspectives of wolffish cultivation by the extraction of high value biomolecules. *In* Seafood quality and safety: Advances in the new millennium. *Edited by* F. Shahidi, and B. K. Simpson. ScienceTech Publishing Co., St John's, N.L. pp. 61–69.

Le François, N. R., Dutil, J. D., Blier, P., Lord, K., and Chabot, D. 2000. Tolerance and growth of juvenile common wolffish (*Anarhichas lupus*) under low salinity and hypoxic conditions: Preliminary results. *Aquaculture Canada 2000 – Aquaculture Association of Canada Special Publication* **4**, 57–59.

Le François, N. R., Lemieux, H., and Blier, P. U. 2002. Biological and technical evaluation of the potential of marine and anadromous fish species for cold-water mariculture. Aquacult. Res. **33**(2): 95–108. doi:10.1046/j.1365-2109.2002.00652.x.

Lewis, R.M. and W.F. Hettler, Jr. 1968. Effect of temperature and salinity on survival of young Atlantic menhaden, *Brevoortia tyrannus*. Estuaries, 10: 347-350.

Llansó, R. J. 1991. Tolerance of low dissolved-oxygen and hydrogen-sulfide by the polychaete *Streblospio-benedicti* (Webster). J. Exp. Mar. Biol. Ecol. **153**: 165–178. doi:10.1016/0022-0981(91)90223-J.

Locke, A., Klassen, G. J., Bernier, R., and Joseph, V. 2005. Life history of the sand shrimp, *Crangon septemspinosa* Say, in a southern Gulf of St. Lawrence estuary. *Journal of Shellfish Research*, **24**(2): 603-613.

Lough, R. G. 2004. Essential fish habitat source document: Atlantic cod, *Gadus morhua*, life history and habitat characteristics. NOAA Tech. Mem. NMFS-NE-190, 94 pp.

Lovrich, G. A., Sainte-Marie, B., and Smith, B. D. 1995. Depth distribution and seasonal movements of *Chionoecetes opilio*(Brachyura: Majidae) in Baie Sainte-Marguerite, Gulf of Saint Lawrence. Can. J. Zool. **73**(9): 1712–1726. doi:10.1139/z95-203.

Lutz, R. A., Goodsell, J. G., Castagna, M., and Stickney, A. P. 1983. Growth of experimentally cultured ocean quahogs (*Arctica islandica* L.) in north temperate embayments. *J. World Maricul. Soc.* **14**(1‐4): 185-190.

MacKenzie, C., and Moring, J. R. 1985. Species Profiles: life Histories and Environmental Requirements of Coastal Fishes and Invertebrates (North Atlantic) – American lobster. U.S. Fish Wildl. Serv. Biol. Rep. 82(11.33). U.S. Army Corps of Engineers, TR EL-82-4. 19 pp.

Magee, J. A., Obedzinski, M., McCormick, S. D., and Kocik, J. F. 2003. 
Effects of episodic acidification on Atlantic salmon (*Salmo salar*) smolts. Can. J. Fish. Aquat. Sci. **60**(2): 214–221. doi:10.1139/F03-015.

Malchoff, M.H. 1993. Age, growth and distribution of cunner (*Tautogolabrus adspersus*) and tautog (*Tautoga onitis*) larvae in the New York Bight: a single season analysis. M.S. thesis, Bard Coll., Annandale-on-Hudson, NY. 75 p.

Mallet, A. L., and Carver, C. E. 1995. Comparative growth and survival patterns of *Mytilus trossulus* and *Mytilus edulis* in Atlantic Canada. *Can. J. Fish. Aquat. Sci.* **52**(9): 1873-1880.

Malloy, K. D., and Targett, T. E. 1991. Feeding, growth and survival of juvenile summer flounder *Paralichthys dentatus*: experimental analysis of the effects of temperature and salinity. *Mar. Ecol. Prog. Ser.* **72**(3): 213-223.

Malone, P. G., and Dodd, J. R. 1967. Temperature and salinity effects on calcification rate in *Mytilus edulis* and its paleoecological implications. Limnol. Oceanogr. **12(**3): 432-436.

Manzi, J. J., and Castagna, M. 1989. Clam mariculture in North America. *Developments in Aquaculture and Fisheries Science. Elsevier, Amsterdam, The Netherlands*.

Marshall, N. 1946. Observations on the comparative ecology and life history of two sea robins, *Prionotus carolinus* and *Prionotus evolans strigatus*. Copeia: 118-144.

Martin, F. D., and Drewry, G. E. 1978. Development of Fishes of the Mid-Atlantic Bight, Volume VI, Stromateidae Through Ogcoephalidae. US Fish Wildl. Serv. FWS/OBS-*78*, 416 pp.

McBride, R. S. 1994. Comparative Ecology and Life History of Two Temperate, Northwestern Atlantic Searobins, *Prionotus Carolinus* and *P. Evolans* (Pisces: Triglidae). Doctoral dissertation, Rutgers University.

McBride, R. S., and Able, K. W. 1994. Reproductive Seasonality, Distribution, and Abundance of *Prionotus carolinus* and *P. evolans* (Pisces: Triglidae) in the New York Bight. Estuar. Coast. Shelf Sci. **38**(2): 173-188.

McCarthy, I., Moksness, E., and Pavlov, D. A. 1998. The effects of temperature on growth rate and growth efficiency of juvenile common wolffish. *Aquacult. Internat.* **6**(3): 207-218.

McCormack, W.H. 1976. Laboratory behavior of young tautog (*Tautoga onitis*) at acclimation temperature and under a temperature increase. M.S. thesis, Long Island Univ., Brookville, NY. 72 p.

McCracken, F. D. 1954. Seasonal movements of the winter flounder, *P. americanus* (Walbaum) on the Atlantic coast. Fish. Res. Bd. Can. MS Rep. Biol. Sta, **582**: 1-167.

McCusker, M. R., and Bentzen, P. 2010. Historical influences dominate the population genetic structure of a sedentary marine fish, Atlantic wolffish (*Anarhichas lupus*), across the North Atlantic Ocean. Mol. Ecol. **19**(19): 4228–4241. doi:10.1111/j.1365-294X.2010.04806.x.

McEachran, J. D., and Musick, J. A. 1975. Distribution and relative abundance of seven species of skates (Pisces: Rajidae) which occur between Nova Scotia and Cape Hatteras. Fish. Bull. **73**(1): 110-136.

McLeese, D. W., and Watson, J. 1968. Oxygen consumption of the spider crab (*Chionoecetes opilio*) and the American lobster (*Homarus americanus*) at a low temperature. J.Fish. Res. Bd. Canada **25**(8): 1729-1732.

McRae, E. D. Jr. 1960. Lobster explorations on continental shelf and slope off northeast coast of the United States. *Comm. Fish. Rev.* **22**, 1–7.

Meldrim, J. W., Gift, J. J., and Petrosky, B. R. 1974. Effect of temperature and chemical pollutants on the behavior of several estuarine organisms. No. PB-239347; BULL-11. Ichthyological Associates, Inc., Middletown, Del. USA.

Melzner, F., Göbel, S., Langenbuch, M., Gutowska, M.A., Pörtner, H.O. and Lucassen, M. 2009. Swimming performance in Atlantic cod (*Gadus morhua*) following long-term (4–12 months) acclimation to elevated seawater PCO2. Aquat. Toxicol., **92**(1): 30-37.

Mendonça, P. C., and Gamperl, A. K. 2010) The effects of acute changes in temperature and oxygen availability on cardiac performance in winter flounder (*Pseudopleuronectes americanus*). Comp. Biochem. Physiol. Part A: Molec. Integrat. Physiol. **155**(2): 245-252.

Mercer, L. P. 1989. Species profiles: life histories and environmental requirements of coastal fishes and invertebrates (Mid-Atlantic) – weakfish. U.S. Fish Wildl. Serv. Biol. Rep. 82(11.109). U.S. Army Corps of Engineers, TR EL-82-4. 17 pp.

Merrill, A. S., and Ropes, J. W. 1969. The general distribution of the surf clam and ocean quahog. In *Proc. Natl. Shellfish. Assoc*. **59**(4): 40-45.

Miller, D. C., Poucher, S. L., and Coiro, L. 2002.  Determination of lethal dissolved oxygen levels for selected marine and estuarine fishes, crustaceans, and a bivalve. Mar. Biol. **140**(2): 287–296. doi: 10.1007/s002270100702.

Mills, K. E., Pershing, A. J., Sheehan, T. F., and Mountain, D. 2013. Climate and ecosystem linkages explain widespread declines in North American Atlantic salmon populations. Glob. Change Biol. doi:10.1111/gcb.12298.

Mistri, M. 2004. Predatory behavior and preference of a successful invader, the mud crab *Dyspanopeus sayi*(Panopeidae), on its bivalve prey. J. Exp. Mar. Biol. Ecol. **312**(2): 385–398. doi:10.1016/j.jembe.2004.07.012.

Moore, E. 1947. Studies on the marine resources of southern New England. VI. The sand flounder, *Lophopsetta aquosa* (Mitchill); a general study of the species with special emphasis on age determination by means of scales and otoliths. Bull. Bingham Oceanogr. Collect. 11(3): 1-79.

Moran, D. and Stottrup, J.G. 2011. The effect of carbon dioxide on growth of juvenile Atlantic cod *Gadus morhua* L. Aquat Toxicol, **102**(1-2): 24-30.

Morin, B., Hudon, C., and Whoriskey, F. 1991. Seasonal distribution, abundance, and life-history traits of Greenland cod, *Gadus ogac*, at Wemindji, eastern James Bay. Can. J. Zoo. **69**(12): 3061-3070.

Morrison, G. 1971. Dissolved oxygen requirements for embryonic and larval development of the hardshell clam, Mercenaria mercenaria. J. Fish. Bd. Canada, **28**(3): 379-381.

Morse, W. W., Johnson, D. L., Berrien, P. L., and Wilk, S. J. 1999. Essential fish habitat source document: silver hake, *Merluccius bilinearis*, life history and habitat characteristics. NOAA Tech. Memo. NMFS-NE-135, Woods Hole, M.A. 42 pp.

Moser, M. L., Ross, S. W., and Sulak, K. J. 1996. Metabolic responses to hypoxia of *Lycenchelys verrillii* (wolf eelpout) and *Glyptocephalus cynoglossus* (witch flounder): sedentary bottom fishes of the Hatteras/Virginia Middle Slope. Mar. Ecol. Prog. Ser. **14**(1): 57-61.

Mucci, A., Starr, M., Gilbert, D. and Sundby, B. 2011. Acidification of Lower St. Lawrence Estuary Bottom Waters. Atmosphere-Ocean, **49**(3): 206-218.

Mullen, D. M., and Moring, J. R. 1986. Species profiles: life histories and environmental requirements of coastal fishes and invertebrates (North Atlantic) – sea scallop. U.S. Fish Wildl. Serv. Biol. Rep. 82(11.67). U.S. Army Corps of Engineers, TR EL-82-4. 13 pp.

Musick, J. A. 1972. Fishes of Chesapeake Bay and the adjacent coastal plain. A checklist of the biota of lower Chesapeake Bay. Va. Inst. Mar. Sci. Spec. Publ. **65**: 175-212.

Narayan, S., Carscadden, J., Dempson, J. B., O'Connell, M. F., Prinsenberg, S., Reddin, D. G., and Shackell, N. 1995. Marine climate off Newfoundland and its influence on salmon (*Salmo salar*) and capelin (*Mallotus villosus*). *In* Climate change and northern fish populations. *Edited by* R. J. Beamish. Canadian Special Publication of Fisheries and Aquatic Sciences no. 121. pp. 461–474.

Newell, R. I. E. 1989. Species profiles: life histories and environmental requirements of coastal fishes and invertebrates (North–Mid-Atlantic) – blue mussel. U.S. Fish. Wildl. Serv.Biol. Rep. 82 (11.102). U.S. Army Corps of Engineers, TR El-82-4, 25 pp.

Newell, C. R., and Hidu, H. 1986. Species profiles: life histories and environmental requirements of coastal fishes and invertebrates (North Atlantic) – softshell clam. U.S. Fish Wildl. Serv. Biol. Rep. 82(11.53). U.S. Army Corps of Engineers, TR EL-82-4. 17 pp.

Niklitschek, E. J., and Secor, D. H. 2009. Dissolved oxygen, temperature and salinity effects on the ecophysiology and survival of juvenile Atlantic sturgeon in estuarine waters: I. Laboratory results. J. Exper. Mar. Biol. Ecol. **381**: S150-S160. doi:10.1016/j.jembe.2009.07.018.

NOAA. 1977. Oxygen depletion and associated environmental disturbances in the Middle Atlantic Bight in 1976. A report on a series of interagency workshops held in November and December 1976. Tech. Ser. Rep. No. 3, Northeast Fisheries Center, national Marine Fisheries Service, NOAA, U.S. Dept. of Commerce, Sandy Hook, N.J. 471 pp.

NODC. 2011. World Ocean Database 2009 [online]. Available from http://www.nodc.noaa.gov/OC5/WOD/pr_wod.html [ accessed 3 July 2013].

O'Dea, N. R., and Haedrich, R. L. 2000. COSEWIC status report on the Atlantic wolffish *Anarhichas lupus*in Canada. [Online]. Committee on the Status of Endangered Wildlife in Canada, Ottawa, O.N. Available from http://www.sararegistry.gc.ca/virtual_sara/files/cosewic/sr_atlantic_wolffish_1100_e.pdf [accessed 16 July 2013].

O'Dea, N. R., and Haedrich, R. L. 2002. A review of the status of the Atlantic wolffish, *Anarhichas lupus*, in Canada. Can. Field Nat. **116**(3): 423–432.

Ouellet, P. and Lefaivre, D. 1994. Vertical distribution of northern shrimp (*Pandalus borealis*) larvae in the Gulf of St Lawrence; implications for trophic interactions and transport. Can. J. Fish. Aquat. Sc., **51**(1): 123-132.

Packer, D. B., Griesbach, S. J., Berrien, P. L., Zetlin, C. A., Johnson, D. L., and Morse, W. W. 1999. Essential fish habitat source document: Summer flounder, *Paralichthys dentatus*, life history and habitat characteristics. NOAA Tech. Memo. NMFS-NE Ser. NMFS-NE-151. 88 pp.

Packer, D. B., and Hoff, T. 1999. Life history, habitat parameters, and essential habitat of mid-Atlantic summer flounder. *In*Am. Fish. Soc. Symp. **22**: 76-92.

Pavlov, D. A., and Novikov, G. G. 1993. Life history and peculiarities of common wolffish (*Anarhichas lupus*) in the White Sea. ICES J. Mar. Sci. **50**(3): 271–277. doi:10.1006/jmsc.1993.1029.

Pearce, J.B. 1969. Thermal addition and the benthos, Cape Cod Canal*.* Chesapeake Sci. **10**:227-233.

Pereira, J. J., Goldberg, R., Ziskowski, J. J., Berrien, P. L., Morse, W. W., and Johnson, D. L. 1999. Essential fish habitat source document: Winter flounder, *Pseudopleuronectes americanus*, life history and habitat characteristics. NOAA Tech. Memo. NMFS-NE, **138**: 1-39.

Peters, D.S. and J.W. Angelovic. 1971. Effect of temperature, salinity, and food availability on growth and energy utilization of juvenile summer flounder, *Paralichthys dentatus*. In D.J. Nelson et, Proc. 3^rd^ Natl. Symp. Radioecology USAEC Conf., -710501-PI. P. 545554. National Technical Information Service, Springfield, VA.

Pierce, G. J., Diack, J. S. W., and Boyle, P. R. 1989. Digestive tract contents of seals in the Moray Firth area of Scotland. J. Fish Biol. **35**(Suppl. A): A341– A343. doi:10.1111/j.1095-8649.1989.tb03081.x.

Plante, S., Chabot, D., and Dutil, J. D. 1998. Hypoxia tolerance in Atlantic cod. J. Fish Biol. **53**(6): 1342-1356.

Pohle, G. W. 1990. A guide to decapod Crustacea from Atlantic Canada: Anomura and Brachyura. Can. Tech. Rep. Fish. Aquat. Sci. **1771**: 1-30.

Posey, M. H., and Hines, A. H. 1991. Complex predator-prey interactions within an estuarine benthic community. Ecology, **72**(6): 2155–2169.

Powles, P. M. 1967. Atlantic wolffish (*Anarhichas lupus*L.) eggs off southern Nova Scotia. J. Fish. Res. Board Can. **24**(1): 207–209.

Purcell, J. E., Malej, A. and Benovic, A. 1999. Potential links of jellyfish to eutrophication and fisheries. *In* Malone, T. C., Malej, A. Harding, L. W. , Jr., Smodlaka, N. and Turner, R. E. (Eds), Ecosystems at the Land–Sea Margin: Drainage Basin to Coastal Sea. American Geophysical Union, Coastal and Estuarine Studies, **55**: 241–263.

Purcell, J. E., Shiganova, T. A., Decker, M. B., and Houde, E. D. 2001. The ctenophore *Mnemiopsis* in native and exotic habitats: U.S. estuaries versus the black sea basin. Hydrobiologia, **451**(1–3):145–176. doi:10.1023/A:1011826618539.

Randall, R. G., Jones, R. P., Minns, C. K., and Rice, J. C. 2005. Proceedings of a case study review of critical habitat identification for aquatic species-at-risk, Bedford Institute of Oceanography, Dartmouth, N.S. Can. Sci. Advis. Sec. Proc. Ser. 2004/047.

Reddin, D. G., and Shearer, W. M. 1987. Sea-surface temperature and distribution of Atlantic salmon in the northwest Atlantic Ocean. *In* Common Strategies of Anadromous and Catadromous Fishes: Proceedings of an International Symposium Held in Boston, Massachusetts, USA, March 9–13 1986. American Fisheries Society Symposium, 1. pp. 262–275.

Remen, M., Oppedal, F., Torgersen, T., Imsland, A. K., and Olsen, R. E. 2012. Effects of cyclic environmental hypoxia on physiology and feed intake of post-smolt Atlantic salmon: Initial responses and acclimation. Aquaculture, **326**–**329**: 148–155. doi:10.1016/j.aquaculture.2011.11.036.

Richards, S. W., Mann, J. M., and Walker, J. A. 1979. Comparison of spawning seasons, age, growth rates, and food of two sympatric species of searobins, *Prionotus carolinus* and *Prionotus evolans*, from Long Island Sound. Estuar. **2**(4): 255-268.

Ries, J. B., Cohen, A. L. and McCorkle, D. C. 2009. Marine calcifiers exhibit mixed responses to CO2-induced ocean acidification. Geology, **37**(12): 1131-1134.

Ristich, S. S., Crandall, M., and Fortier, J. 1977. Benthic and epibenthic macroinvertebrates of the Hudson River: I. Distribution, natural history and community structure. Estuar. Coast. Mar. Sci. **5**(2): 255-266.

Rodger, R. W. A., and von Zharen, W. M. 2011. Introduction to the commercial fisheries of the United States and Canada. Canadian Marine Publications, Halifax, N.S.

Roff, J. C., Fanning, L. P., and Stasko, A. B. 1984. Larval crab (*Decapoda: Brachyura*) zoeas and megalopas of the Scotian Shelf. Can. Tech. Rep. Fish. Aquat. Sci. **1264**: 22 pp.

Rogers, S. G., and Van Den Avyle, M. J. 1989. Species profiles: life histories and environmental requirements of coastal fishes and invertebrates (Mid-Atlantic) – Atlantic menhaden. US Fish Wildl. Serv. 82 (11.108). U.S. Army Corps of Engineers TR EL-82-4. 23 pp.

Sandifer, P. A. 1975. The role of pelagic larvae in recruitment to populations of adult decapod crustaceans in the York River estuary and adjacent lower Chesapeake Bay, Virginia. Estuarine and Coastal Marine Science, **3**(3): 269-279.

Schwartz, F. J. 1963. The barrelfish from Chesapeake Bay and the Middle Atlantic bight, with comments on its zoogeography. Chesapeake Science, **4**(3): 147-149.

Scott, J. S. 1982. Depth, temperature and salinity preferences of common fishes of the Scotian Shelf. J. Northwest Atl. Fish. Sci, **3**(1): 29-39.

Scott, D. 2001. Chemical pollution as a factor affecting the sea survival of Atlantic salmon, *Salmo salar*L. Fish. Manage. Ecol. **8**(6): 487–499. doi:10.1046/j.1365-2400.2001.00277.x.

Scott, W. B., and Scott, M. G. 1988. Atlantic fishes of Canada. Can. Bull. Fish. Aquat. Sci: **219**: 731p.

Sellers, M. A., and Stanley, J. G. 1984. Species Profiles: Life Histories and Environmental Requirements of Coastal Fishes and Invertebrates (North Atlantic) – American oyster. US Fish Wildl. Serv. FWS/OBS-82/11.23. U.S. Army Corps of Engineers TR EL-82-4. 15 pp.

Setzler, E. M., Boynton, W. R., Wood, K. V., Zion, H. H., Lubbers, L., Mountford, N. K. et al. 1980. Synopsis of biological data on striped bass, *Morone saxatilis* (Walbaum). NOAA Tech. Rep. NMFS Circular 433.

Shimps, E. L., Rice, J. A., and Osborne, J. A. 2005. Hypoxia tolerance in two juvenile estuary-dependent fishes. J. Exper. Mar. Biol. Ecol. **325**(2): 146-162.

Shumway, S. E., Perkins, H. C., Schick, D. F., and Stickney, A. P. 1985. Synopsis of biological data on the pink shrimp, *Pandalus borealis* Krøyer, 1838. FAO Fisheries Synopsis, 144; NOAA Technical Report NMFS, 30: 57 p.

Sibunka, J. D., and Pacheco, A. L. 1981. Biological and fisheries data on northern puffer, *Sphoeroides maculatus* (Bloch and Schneider). Northeast Fisheries Center, Tech. Ser. Rep. No. 26. 56 pp.

Skaphandrus. [online]. Available from http://skaphandrus.com.

Squires, H. J. 1990. Decapod Crustacea of the Atlantic coast of Canada. Bull. Fish. Aquat. Sci. **221**: 532 pp.

Stanley, J. G., and DeWitt, R. (1983). Species Profiles: life Histories and environmental requirements of coastal fishes and invertebrates (North Atlantic) – hard clam. U.S. Fish Wildl. Serv. FWS/OBS-82/11.18. U.S. Army Corps of Engineers TR EL-82-4. 19 pp.

Steffensen, J. F., Bushnell, P. G., and Schurmann, H. 1994. Oxygen consumption in four species of teleosts from Greenland: no evidence of metabolic cold adaptation. Polar Biol. 14(1): 49-54.

Steimle, F. W., and Shaheen, P. A. 1999. Essential fish habitat source document: Tautog (*Tautoga onitis*) life history and habitat requirements. NOAA Technical Memorandum NMFS-NE-118. U.S. Department of Commerce, Washington, D.C.

Steimle, F. W., and Sinderman, C. J. 1978. Review of oxygen depletion and associated mass mortalities of shellfish in the Middle Atlantic Bight in 1976. Mar. Fish. Rev. **40**(12): 17-26.

Steimle, F. W., Morse, W. W., Berrien, P. L., and Johnson, D. L. 1999. Essential fish habitat source document: red hake, *Urophycis chuss*, life history and habitat characteristics. NOAA Tech. Memo. NMFS-NE-133, 34 pp.

Stevens, E. D., Sutterlin, A., and Cook, T. 1998. Respiratory metabolism and swimming performance in growth hormone transgenic Atlantic salmon. Can. J. Fish. Aquat. Sci. **55**(9): 2028–2035. doi:10.1139/f98-078.

Stevenson, D. K., and Scott, M. L. 2005. Essential fish habitat source document: Atlantic herring, *Clupea harengus*, life history and habitat characteristics. NOAA Tech. Memo. NMFS-NE-192, 84 pp.

Stewart, P. L., and Arnold, S. H. 1994. Environmental requirements of the sea scallop (*Placopecten magellanicus*) in eastern Canada and its response to human impacts. Can. Tech. Rep. Fish. Aquat. Sci. 2005: 36 pp.

Stickle, W. B., Kapper, M. A., Liu, L.-L., Gnaiger, E., and Wang, S. Y. 1989. Metabolic adaptations of several species of crustaceans and molluscs to hypoxia: Tolerance and microcalorimetric studies. Biol. Bull. **177**(2): 303–312.

Steneck, R. S., and Wilson, C. J. 2001. Large-scale and long-term, spatial and temporal patterns in demography and landings of the American lobster, *Homarus americanus*, in Maine. Mar. Freshwater Res. **52**: 1303-1319.

Stone, H. H., and Jessop, B. M. 1992. Seasonal distribution of river herring *Alosa pseudoharengus* and *A. aestivalis* off the Atlantic coast of Nova Scotia. Fish. Bull. **90**(2): 376-389.

Studholme, A. L., Packer, D. B., Berrien, P. L., Johnson, D. L., Zetlin, C. A., and Morse, W. W. 1999. Essential fish habitat source document: Atlantic mackerel, *Scomber scombrus*, life history and habitat characteristics. NOAA Tech. Memo. NMFS-NE-141, 35 pp.

Szedlmayer, S. T., and Able, K. W. 1993. Ultrasonic telemetry of age-0 summer flounder, *Paralichthys dentatus*, movements in a southern New Jersey estuary. Copeia: 728-736.

Tagatz, M. E. 1967. Noncommercial crabs of the genus *Callinectes* in St. Johns River, Florida. Chesapeake Science, **8**(3): 202-203.

Tagatz, M. E., and Dudley, D. L. 1961. Seasonal occurrence of marine fishes in four shore habitats near Beaufort, NC, 1957-1960. U.S. Fish and Wildlife Service, Special Scientific Reports, Fisheries **390**, 19 pp.

Targett, T. E. 1979. The effect of temperature and body size on digestive efficiency in *Fundulus heteroclitus* (L.). J. Exper. Mar. Biol. Ecol. **38**(2): 179-186.

Taylor, A. C., and Brand, A. R. 1975. A comparative study of the respiratory responses of the bivalves *Arctica islandica* (L.) and *Mytilus edulis* L. to declining oxygen tension. Proc. R. Soc. Lond. B. **190**(1101): 443-456.

Templeman, W. 1937. Egg-laying and hatching postures and habits of the American lobster. J. Biol. Bd. Can. **2**: 223-226.

Templeman, W. 1984. Migrations of wolffishes, *Anarhichas* sp., from tagging in the Newfoundland area. J. Northwest Atl. Fish. Soc. **5**(1): 93–97.

Theede, H., Ponat, A., Hiroki, K., and Schlieper, C. 1969. Studies on the resistance of marine bottom invertebrates to oxygen-deficiency and hydrogen sulphide. Marine Biology, **2**(4): 325-337.

Thomas, D. L. 1971. An ecological study of the Delaware River in the vicinity of Artificial Island. Part III. The early life history and ecology of six species of drum (Sciaenidae) in the lower Delaware River, a brackish tidal estuary. Ichthyological Ass. Bull. No. 3, 247 pp.

Thorarensen, H., Gústavsson, A., Mallya, Y., Gunnarsson, S., Árnason, J., Arnarson, I., and Imsland, A. K. 2010. The effect of oxygen saturation on the growth and feed conversion of Atlantic halibut (*Hippoglossus hippoglossus* L.). Aquaculture, **309**(1): 96-102.

U.S. Geological Survey. 2013. Dissolved oxygen solubility tables [online]. Available from http://water.usgs.gov/software/DOTABLES/ [accessed 22 May 2013].

Van Dam, L. 1935. On the utilization of oxygen by Mya arenaria. J. Exp. Biol. **12**: 86:94.

Vernberg, F. J., and Vernberg, W. B. 1975. Adaptations to extreme environments. In: Verberg, F. J. (ed.) Physiological ecology of estuarine organisms. University of South Carolina Press, Columbia, 165-180.

Voyer, R. A., and Hennekey, R. J. 1972. Effects of dissolved oxygen on two life stages of the mummichog.  Progr. Fish-Cultur. **34**(4): 222-225.

Weiss-Glanz, L. S., Stanley, J. G. and Moring, J. R. 1986. Species Profiles: life histories and environmental requirements of coastal fishes and invertebrates (North Atlantic)— American shad. U.S. Fish Wildl. Serv. Biol. Rep. 82(11.59). U.S. Army Corps of Engineers, TR EL-82-4, 16 pp.

Whitehead, P. J. P. 1985a. FAO Species Catalogue, Vol. 7. Clupeoid fishes of the world (suborder Clupeodei). Part 1. Chirocentridae, Clupeidae and Pristigasteridae. Food and Agriculture Organization of the United Nations (FAO) Fish Synop. No. 125, **7**(1): 1-303.

Whitehead, P. J. P. 1985b. King Herring: his place amongst the clupeoids. Canadian J. Fish. Aquat. Sci. **42**(S1): s3-s20.

Wilk, S. J., MacHaffie, E. M., McMillan, D. G., Pacheco, A. L., Pikanowski, R. A., and Stehlik, L. L. 1996. Fish, megainvertebrates, and associated hydrographic observations collected in the Hudson-Raritan Estuary, January 1992-December 1993. U.S. Natl. Mar. Fish. Serv. NFS 96-14. 95 pp.

Williams, A. B. 1965. Marine decapod crustaceans of the Carolinas. Fish. Bull. U.S. Fish Wildl. Serv. **65**: 56-60.

Williams, A. B. 1984. Shrimps, Lobsters, and Crabs of the Atlantic Coast of the Eastern United States, Maine to Florida. Smithsonian Institution Press, Washington, D. C.

Witting, D. A., and Able, K. W. 1995. Predation by sevenspine bay shrimp *Crangon septemspinosa*on winter flounder *Pleuronectes americanus*during settlement: Laboratory observations. Mar. Ecol. Prog. Ser. **123**: 23–31.

Youcef, W.A., Lambert, Y. and Audet, C. 2013. Spatial distribution of Greenland halibut *Reinhardtius hippoglossoides* in relation to abundance and hypoxia in the estuary and Gulf of St. Lawrence. Fish. Oceanogr., **22**(1): 41-60.

Young, J.S. and C.I. Gibson. 1973. Effect of thermal effluent on migrating menhaden. Mar. Poll. Bull. 4:94-96.
